# Interleukin-27 promotes autophagy in human serum-induced primary macrophages via an mTOR- and LC3-independent pathway

**DOI:** 10.1038/s41598-021-94061-3

**Published:** 2021-07-21

**Authors:** Sylvain Laverdure, Ziqiu Wang, Jun Yang, Takuya Yamamoto, Tima Thomas, Toyotaka Sato, Kunio Nagashima, Tomozumi Imamichi

**Affiliations:** 1 Laboratory of Human Retrovirology and Immunoinformatics, Frederick National Laboratory for Cancer Research, Building 550, Room 126, P.O. Box B, Frederick, MD 21702 USA; 2 Electron Microscope Laboratory, Frederick National Laboratory for Cancer Research, Frederick, MD 21702 USA; 3 Laboratory of Immunosenescence, National Institutes of Biomedical Innovation, Health and Nutrition, Osaka, 567-0085 Japan; 4 Laboratory of Aging and Immune Regulation, Graduate School of Pharmaceutical Sciences, Osaka University, Osaka, 565-0871 Japan

**Keywords:** Cytokines, Interleukins, Macroautophagy, Autophagy

## Abstract

Interleukin-27 (IL-27) is a cytokine that suppresses human immunodeficiency virus (HIV)-1 infection in macrophages and is considered as an immunotherapeutic reagent for infectious diseases. It is reported that IL-27 suppresses autophagy in Mycobacterium tuberculosis-infected macrophages; however, a role for IL-27 on autophagy induction has been less studied. In this study, we investigated the impact of IL-27 in both autophagy induction and HIV-1 infection in macrophages. Primary human monocytes were differentiated into macrophages using human AB serum (huAB) alone, macrophage-colony stimulating factor (M-CSF) alone, or a combination of IL-27 with huAB or M-CSF. Electron microscopy and immunofluorescence staining demonstrated that a 20-fold increase in autophagosome formation was only detected in IL-27 + huAB-induced macrophages. Western blot analysis indicated that the autophagosome induction was not linked to either dephosphorylation of the mammalian target of rapamycin (mTOR) or lipidation of microtubule-associated protein 1A/1B-light chain 3 (LC3), an autophagosomal marker, implying that IL-27 can induce autophagy through a novel non-canonical pathway. Here we show for the first time that IL-27 induces autophagy during monocyte-to-macrophage differentiation in a subtype-dependent manner.

## Introduction

Interleukin (IL)-27—a member of the IL-6/IL-12 family—is a heterodimeric cytokine composed of Epstein-Barr virus-induced gene 3 (EBi3) and IL-27 p28^1^ and is produced from macrophages and dendritic cells upon stimulation^2^. It plays an important role in modulating inflammation in innate and adaptive immune cells as a pro- or anti-inflammatory cytokine^3^. It binds to the specific IL-27 receptor that is comprised of IL-27 receptor subunit (WSX1) and signal transducer glycoprotein 130 (gp130), and activates Janus kinase/signal transducer and activator of transcription protein 3 (JAK/STAT3) pathway^4^. Furthermore, it promotes transforming growth factor-β-activated kinase 1/mitogen-activated protein kinases (TAK1/MAPK) signaling pathway in macrophages^5^. The JAK/STAT3 signal induces multiple functions in various cell types^3^; it can induce proliferation of CD4+ T cells, polarization of Th1 cells, activation of cytotoxic T cells, generation of natural killer cells, and differentiation of macrophages and dendritic cells. Various studies have revealed that IL-27 can inhibit certain subtypes of T cells (Th, Th2 and Th17 cells) by inducing IL-10, increase the expression of PDL1 on immune and non-immune cells, and regulates Treg development or conversion^2^. Not only does IL-27 modulate the function of immune cells, but it also suppresses replication of viruses (e.g. human immunodeficiency virus (HIV)-1 and herpes simplex virus) in the infected cells by directly regulating the host cell function^5^; thus, IL-27 is considered to be a new agent for immunotherapy^6,7^, based on its dual function on both innate and adaptive immune compartments^8,9^.

Macroautophagy (hereafter referred to as autophagy) is a cellular catabolic process that mediates degradation of cytoplasmic constituents such as organelles or protein aggregates by sequestering them into double membrane organelles named autophagosomes before subsequent delivery to lysosomes for degradation, thereby forming autolysosomes; a process of fusion of the multivesicular endosome with the autophagosome to form the amphisomes has also been described^10^. Autophagosome formation is a complex process involving approximately 20 autophagy-related proteins, and the molecular mechanisms of autophagosome induction and formation are well documented. Apart from bearing an essential cellular function for cell survival and differentiation, as well as for development and homeostasis, autophagy can also dispose of some intracellular pathogens, and has therefore been associated with innate immune responses^11^. Control of autophagy activity has the potential for improving therapies for cancer, inflammation and infectious diseases^12,13^.

Autophagy can be regulated by numerous factors such as nutrient signaling, energy sensing, oxidative stress, DNA damage, hypoxia, and generally cellular stress. Moreover, autophagy is also an immunological process used to directly degrade intracellular pathogens (xenophagy)^14^. The infection and replication of HIV-1, a viral pathogen invasion, regulates autophagy induction in infected T cells and macrophages as well as bystander cells^15^. Overall, it has been reported that HIV infection inhibits autophagy induction^16^, while in contrast, the induction of autophagy inhibits HIV replication^17,18^; however, the early, nondegradative stages of autophagy have been shown to promote productive HIV replication in macrophages^18^ and the later stages of autophagy restricts HIV infection by degradation of a viral protein in T cells^19^. These reports indicate that the mechanism by which HIV replication is regulated by autophagy is cell-type dependent. Despite the fact that IL-27 inhibits HIV infection in primary monocyte-derived macrophages^5^, the impact of IL-27 on autophagy induction in macrophages is unclear. In this study, we investigated the role of IL-27 on autophagosome formation in macrophages and report that IL-27 induces autophagy in a macrophage subtype-dependent manner, and that autophagy induction occurs via a mammalian target of rapamycin (mTOR)- and microtubule-associated protein 1A/1B-light chain 3 (LC3)-independent mechanism.

## Results

### HIV-1 infection is inhibited in IL-27 and human serum-induced macrophages

**Figure 1:**
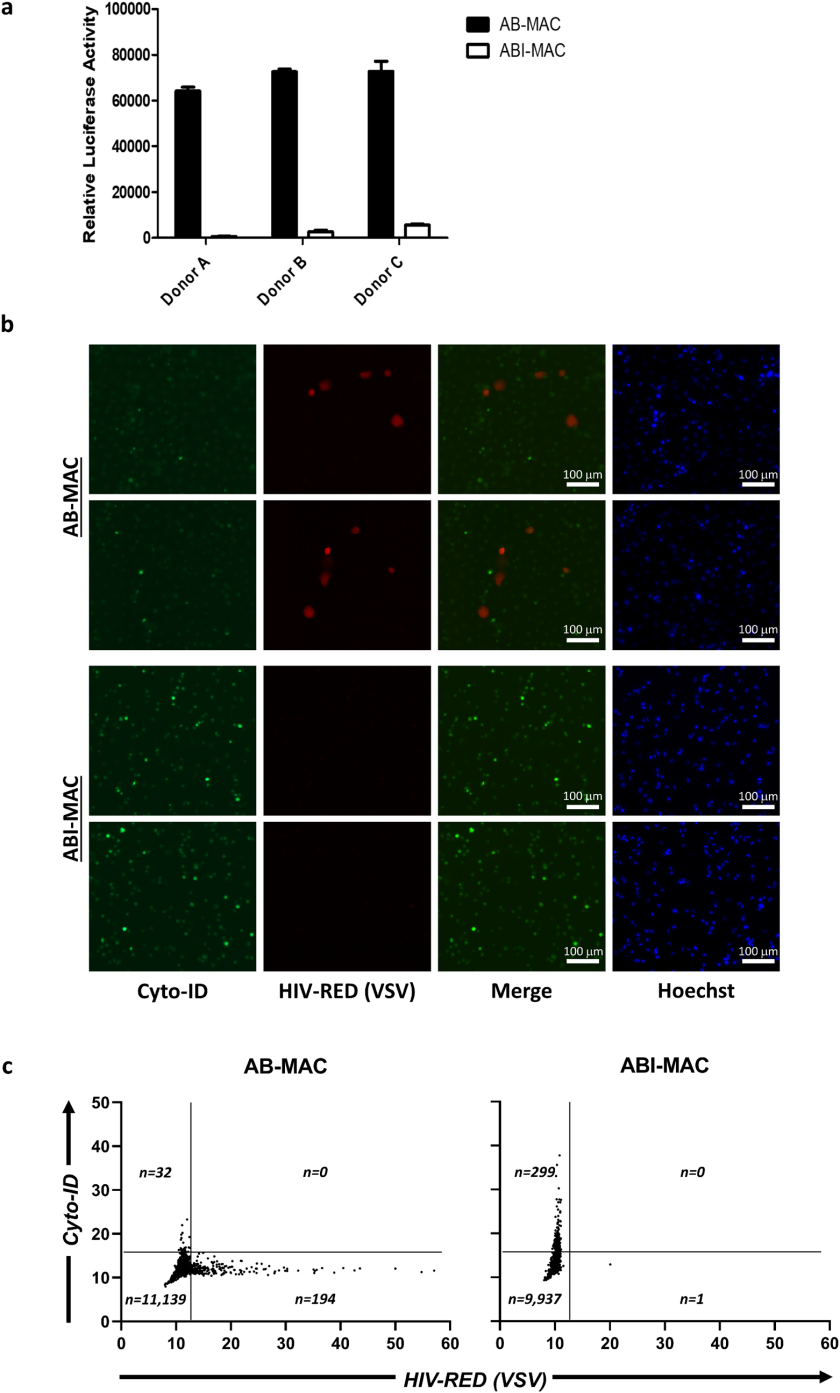
IL-27 treatment during monocyte-to-macrophage differentiation inhibits HIV-1 replication. (**a**) Luciferase activities from AB-MAC and ABI-MAC from three independent donors are represented with solid bars and transparent bars, respectively. Cells were infected with HIVLuc-V and cultured for 24 h. Displayed values are mean relative luciferase activities from three independent experimental triplicates; error bars denote standard deviations. (**b**) AB-MAC and ABI-MAC were infected with HIV-RED-V; 24 h post-infection, cells were stained using the Cyto-ID reagent and Hoechst 33342 as a counterstain. HIV-infected cells are red, autophagic compartments appear green. Pictures display results from one representative donor from three independent assays. (**c**) Single-cell level analysis of HIV-RED and Cyto-ID staining from AB-MAC (left panel) and ABI-MAC (right panel). DIC-based image segmentation was performed on three 2 × 3 composite images at a ×10 magnification for each experimental condition. Dot plots show results from one representative donor and display 11,365 and 10,237 cells for AB-MAC and ABI-MAC, respectively. Population in each quadrant are indicated.

For in vitro macrophage studies, monocytes are differentiated into macrophages using either macrophage-colony stimulating factor (M-CSF) or Granulocyte–Macrophage Colony Stimulating Factor. We previously reported that IL-27 treatment of M-CSF-induced monocyte-derived macrophages triggers resistance to HIV infection, by inhibiting at a post-entry level of the infection of a VSV-G-pseudotyped luciferase-expressing HIV-1 virus (HIVLuc-V)^5^. Although M-CSF is widely used for monocyte differentiation, we decided to confirm our previous findings using a more physiologically relevant cellular model by using human AB serum (huAB) instead throughout this study. Primary human monocytes were differentiated for seven days using either 10% huAB-supplemented D10 medium, creating huAB-induced macrophages (AB-MAC), or a combination of huAB and IL-27, thus creating huAB/IL-27–induced macrophages (ABI-MAC) (Supplementary Fig. S1). We first sought to establish whether IL-27 retained its HIV-1 restriction properties in ABI-MAC using HIVLuc-V. Our results showed that luciferase activity from ABI-MAC was significantly reduced displaying a 95% to 99% reduction (p < 0.0001, n = 3, each donor) when compared to AB-MAC, with minor donor variability (Fig. 1a). These data confirm that IL-27 can induce HIV-resistant cells in huAB-differentiated macrophages, consistent with previous findings using M-CSF-induced macrophages.

Given that multiple reports have linked HIV-1 inhibition to the triggering of autophagy^17,18,20–22^ and because IL-27 has recently been described to suppress Interferon gamma-induced autophagy function in macrophage^23^, we then decided to investigate whether HIV-1 restriction in ABI-MAC could be linked to autophagy induction; we therefore infected both AB-MAC and ABI-MAC with a red fluorescence protein (Ds-RED)-expressing pseudotyped HIV-1 virus (HIV-RED-V), allowing live imaging of infected cells, and simultaneously stained autophagic compartments using Cyto-ID, a recently developed cationic amphiphilic tracer dye which accumulates in autophagic vacuoles^24,25^. As suggested by previous experiments using HIVLuc-V, these results indicated that that HIV-1 replication was restricted in ABI-MAC (Fig. 1b). Interestingly, Cyto-ID staining demonstrated a dramatic accumulation of autophagosomes in ABI-MAC, whereas autophagic material in AB-MAC remained minimal. Using a DIC-based image segmentation approach, we developed a protocol to compare HIV-RED-V and Cyto-ID expression at the single-cell level; through this method, our results demonstrated that HIV-1 expression and autophagy induction were mutually exclusive in our cellular model (Fig. 1c, Supplementary Fig. S2). Indeed, in AB-MAC, every single HIV-RED–positive cell lacked any Cyto-ID staining, while conversely no HIV-RED could be seen in low-level autophagosome-positive cells. A similar trend was seen in ABI-MAC, in which the vast majority of cells were only positive for autophagosome staining. Intriguingly, we detected a few HIV-RED-positive cells among these IL-27–treated cells, all of which lacked autophagosome expression. Overall, our data indicate that IL-27–induced autophagy may restrict HIV-1 infection in ABI-MAC.

### IL-27 triggers autophagy during monocyte-to-macrophage differentiation

**Figure 2:**
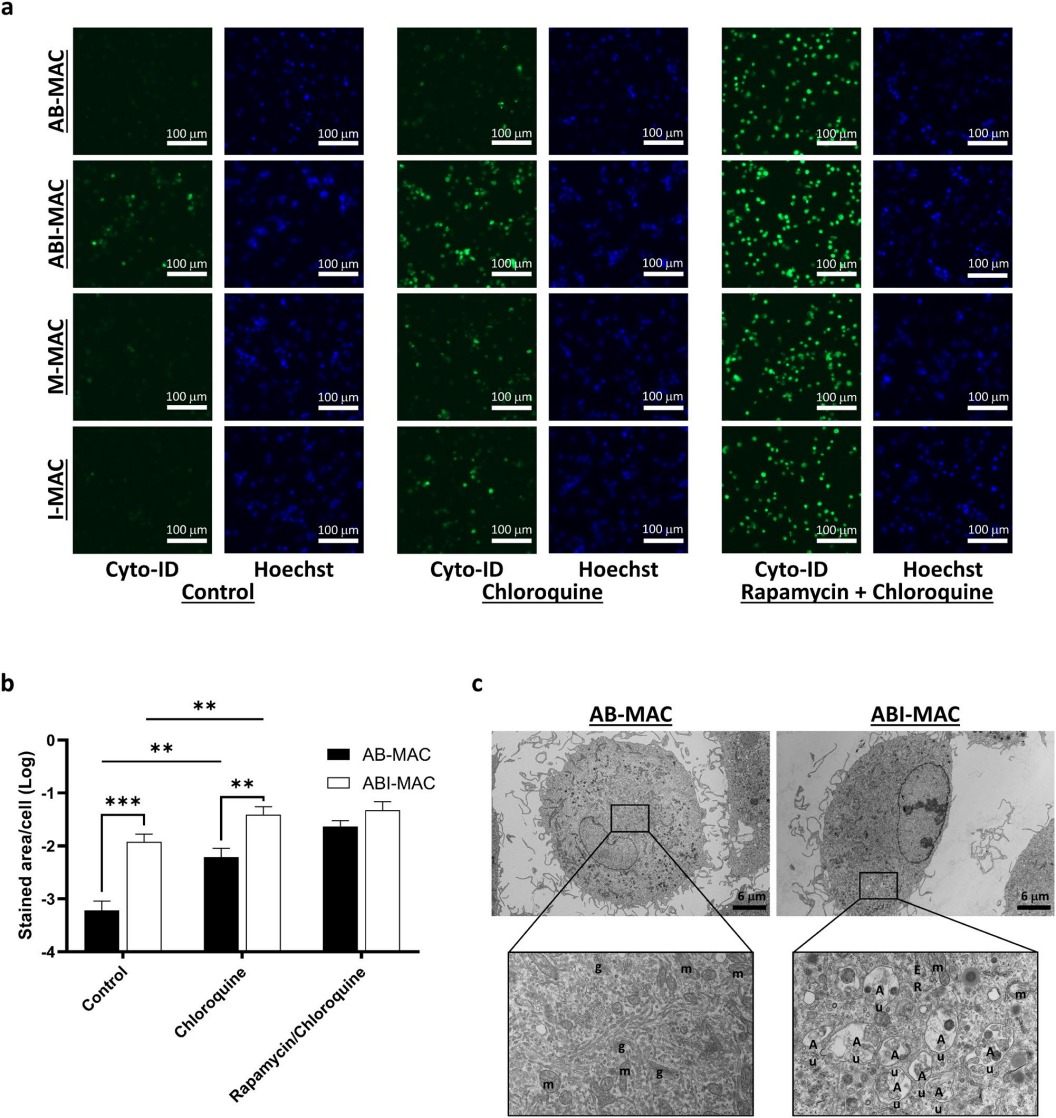
IL-27 induces autophagy in ABI-MAC. (**a**) AB-MAC, ABI-MAC, M-CSF-MAC and IL27 + M-CSF-MAC were incubated overnight in 96-well plate with or without treatment with either chloroquine (10 μM) or a combination of chloroquine and rapamycin (500 nM). Autophagosome staining was then performed using the Cyto-ID reagent, and Hoechst 33342 as a counterstain. 2 × 2 composite images were taken on a Zeiss AxioObserver motorized microscope at a ×10 magnification. (**b**) Autophagy induction in AB-MAC (closed bars) and ABI-MAC (opened bars) was measured. The average stained area per cell for each condition was calculated; values represent means and SD from five independent experiments. Results of significant and relevant Student’s *t*-test are indicated, **p < 0.01, and ***p < 0.001. (**c**) AB-MAC and ABI-MAC were seeded in 6-well plate (1.5 × 10^6^ cells/well) and fixed overnight with 2% glutaraldehyde in cacodylate buffer before being processed and embedded in epoxy resin for TEM imaging as described in the Materials and Methods. Boxed ROIs were magnified for organelle analysis. *g* Golgi, *m* mitochondria, *ER* endoplasmic reticulum, *Au* autophagosome.

Since our initial results indicated an increased autophagic activity in ABI-MAC, we next sought to characterize the autophagic flux in both cell types. As described above, in our previous work, we reported that IL-27 triggered M-CSF-differentiated macrophages into HIV-resistant macrophage^5^; we thus included the IL-27 + M-CSF-induced macrophages and M-CSF per se-induced macrophages as a control. All cell types were incubated overnight in complete medium with or without 10 μM chloroquine, which inhibits lysosome-autophagosome fusion and hence blocks the autophagic flux, or a combination of chloroquine and 500 nM rapamycin (an mTOR-mediated autophagy inducer) as a positive control. Subsequently, autophagosome staining was carried out using the Cyto-ID reagent and Hoechst 33342 as a counterstain (Fig. 2a). As reported hitherto, we found that autophagy was only upregulated in ABI-MAC when compared to other cell types; interestingly, autophagy induction was similar for both M-CSF-induced macrophages, with or with IL-27 treatment. Moreover, all cell types showed an increased autophagosome staining upon chloroquine treatment, indicating that autophagosomes were actively being formed in both cell types, and suggesting that the increased Cyto-ID staining observed in ABI-MAC was due to a faster accumulation rate rather than a degradation inhibition. Of note, all cell types exhibited an intense autophagosome staining when treated with the chloroquine-rapamycin combination.

In order to quantify differences in autophagy staining, we developed a two-channel image analysis pipeline allowing us to account for both cell count and autophagy staining from composite images (Supplementary Fig. S3), thus retrieving an average autophagy staining/cell ratio, which was used to compare autophagy induction among our experimental conditions. Using this method, we found a 20-fold autophagic compartment increase in ABI-MAC when compared to AB-MAC (*p* = 0.0008, n = 5) (Fig. 2b). Similarly, chloroquine-treated cells displayed the same autophagy-increasing trend with ABI-MAC, showing a sixfold increase over AB-MAC (*p* = 0.0018, n = 5). In addition, as suggested in Fig. 2a, both cell types exhibited an active autophagic flux, as demonstrated through tenfold (*p* = 0.0022, n = 5) and threefold (*p* = 0.0061, n = 5) autophagosome enrichments following chloroquine treatment for AB-MAC and ABI-MAC, respectively. Of note, the autophagy induction by chloroquine + rapamycin treatment in ABI-MAC was comparable to that in AB-MAC. We then used transmitted electron microscopy to further confirm autophagosome enrichment in ABI-MAC. Both cell types were differentiated as described above, then fixed overnight in glutaraldehyde before being processed and embedded in epoxy resin. As shown in Fig. 2c, both cell types showed abundant mitochondria, endoplasmic reticulum and lysosomes, consistent with their macrophage phenotype; however, for ABI-MAC, a large number of autophagosomal structures were identified in the perinuclear area, displaying various stages of autophagic degradation. Although we could not ascertain the specific content in autophagic vacuoles, both early initial autophagic vacuoles, presenting intact cytoplasm and double layer membranes, and late degradative vacuoles could be identified in ABI-MAC. Similar to results obtained using the Cyto-ID reagent, these structures were enriched in both AB-MAC and ABI-MAC when treated with chloroquine prior to fixation and embedding (Supplementary Fig. S4)**.** Overall, this further supports the notion that IL-27 induces autophagy in ABI-MAC.

To define the molecular mechanism of the autophagy induction in ABI-MAC, gene expression analysis was performed using Affymetrix Human GeneArray 2.0 microarray chips with AB-MAC and ABI-MAC mRNA samples from three independent donors. The expression level of autophagy-related genes (ATGs) in ABI-MAC was compared to that in AB-MAC. A total of 1561 ATGs listed in three autophagy data bases (Human Autophagy Moderator database, Human Autophagy Database, and Autophagy Database) were analyzed and a total of 734 ATGs were detected in both cell types; however, none of those genes were differentially upregulated in ABI-MACs by more than 3-fold, compared to AB-MAC (Supplementary Table S1). The expression of cyclin-dependent kinase 1 (CDK1) was downregulated in ABI-MAC by 3.2-fold (*p* < 0.05) compared to that in AB-MAC; however, CDK1 is known to positively regulate autophagy and it is reported that a direct or indirect down-regulation of CDK1 reduces autophagy^26–28^. Therefore, it is plausible that this suppression of CDK1 in ABI-MAC may not be associated with the induction of autophagy. Overall, these results indicate that the enhanced autophagic activity seen in ABI-MAC is not regulated at the transcriptional level.

### IL-27-induced autophagy is non-canonical

**Figure 3:**
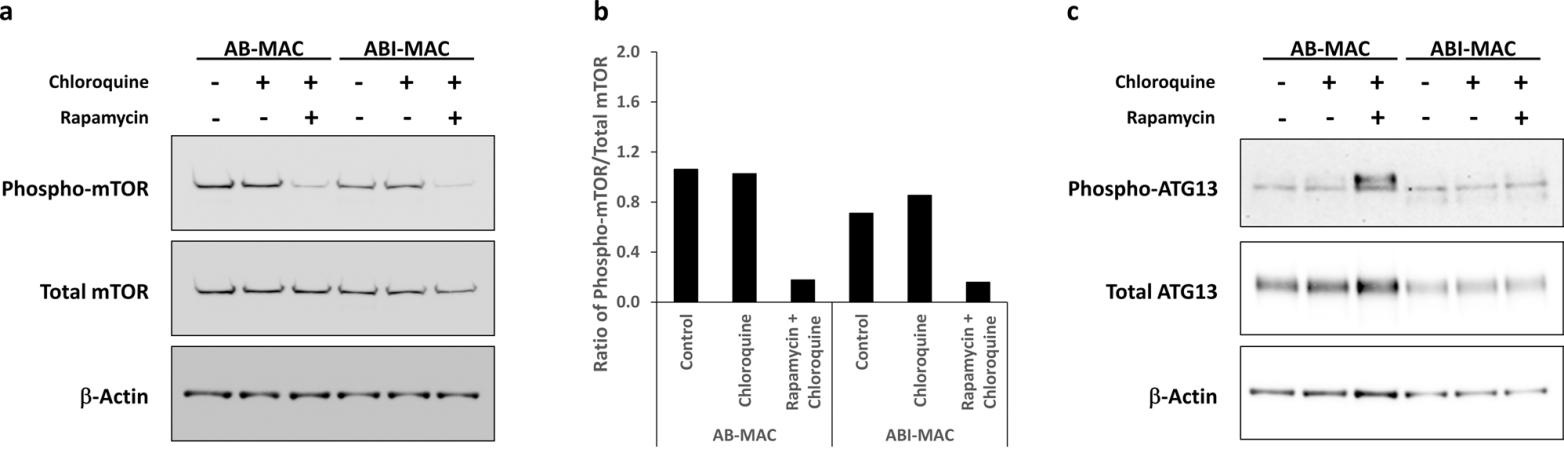
Induction of autophagy by IL-27 is independent of mTOR activity. AB-MAC and ABI-MAC were seeded in 6-well plates (1.5 × 10^6^ cells /well) and incubated overnight in complete medium with or without either chloroquine or a combination of chloroquine and rapamycin as described in the Materials and Methods. (**a**) Total cell lysates were separated on a 3–8% Tris–Acetate gel and transferred onto a 0.45 μm nitrocellulose membrane, which was cut into two pieces along the 52 kDa marker, and phospho-mTOR Ser2448 and β-Actin were detected by western blot. The phospho-mTOR antibody was then stripped from the membrane using a stripping buffer (Thermo Fisher), and the membrane was re-probed with anti-total mTOR antibody. Results display representative blots from a single donor, original blot images are displayed in Supplementary Fig. S9. (**b**) Densitometry analysis of mTOR phosphorylation ratio was performed using the NIH ImageJ software; graph displays densitometry ratios of phospho-mTOR to total mTOR. Representative blots from a single donor. (**c**) Cell lysates were subsequently run on a 4–12% Bis–Tris gel in MOPS buffer (Thermo Fisher), transferred onto a 0.45 μm nitrocellulose membrane, then phospho-ATG13 Ser355 was detected by western blot. The antibody was then stripped from the membrane, which was subsequently probed for total ATG13 and β-Actin. Original blot images are displayed in Supplementary Fig. S10.

To characterize the mechanism of the autophagosome formation in ABI-MAC, both AB-MAC and ABI-MAC were treated for 16 h with either chloroquine or a combination of chloroquine and rapamycin, whereas a subset of each cell type was left untreated as a control. Cells were subsequently lysed, and the samples were subjected to western blot analysis. Because inhibition of the autophagosome preinitiation complex is regulated by the mTOR protein, which maintains this complex in an inactivated state through its serine/threonine kinase activity^29,30^, we first decided to investigate mTOR phosphorylation status. Surprisingly, we found that mTOR phosphorylation levels were similar when comparing untreated AB-MAC and ABI-MAC (Fig. 3a). In fact, densitometry analysis of phosphorylated mTOR ratios across all experimental samples (Fig. 3b) revealed that both untreated control samples and chloroquine-treated samples were displaying the same mTOR phosphorylation levels for both AB-MAC and ABI-MAC. As expected, samples treated with rapamycin exhibited reduced phosphorylation levels in both cell types. This indicated that autophagy induction in ABI-MAC was in fact not linked to mTORC1 inhibition. Since downstream signaling of mTOR involves ATG13, we also investigated ATG13 phosphorylation status following IL-27 treatment (Fig. 3c). Interestingly, we found that basal phosphorylation levels of ATG13 Ser355 in AB-MAC and ABI-MAC were similar; however, while ATG13 was phosphorylated in the presence of rapamycin in AB-MAC, this phosphorylation was inhibited in IL-27-treated cells. This result is particularly striking, considering that ABI-MAC do respond to rapamycin treatment by increasing autophagy levels. This would suggest that while mTORC1 phosphorylation of ATG13 is inhibited in ABI-MAC, mTOR-mediated induction of autophagy is still functional.

**Figure 4:**
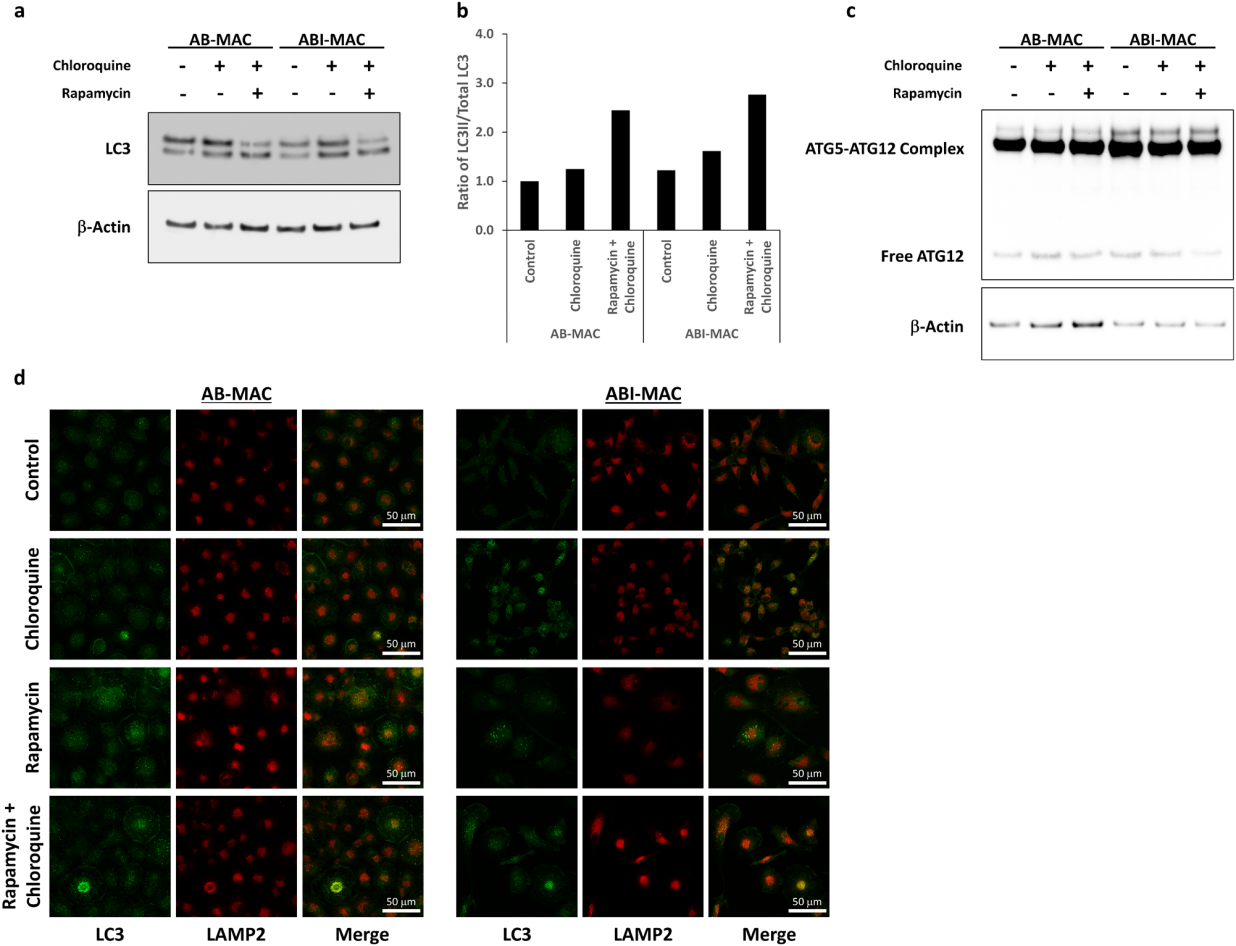
IL-27–induced autophagy is LC3-independent. AB-MAC and ABI-MAC were seeded in 6-well plates (1.5 × 10^6^ cells /well) and incubated overnight in complete medium with or without either chloroquine or a combination of chloroquine and rapamycin as described previously. (**a**) Total cell lysates were separated on a 4–12% Bis–Tris gel in MES buffer (Thermo Fisher) and transferred onto a 0.2 μm nitrocellulose membrane, which was cut into two pieces along the 31 kDa marker. LC3 and β-Actin were detected by western blot. Results display representative blots from a single donor; LC3-I corresponds to the diffuse, cytoplasmic form of LC3, while LC3-II is the lipidated, phosphatidylethanolamine-coupled form of LC3, incorporated into autophagosomes. Original blot images are displayed in Supplementary Fig. S11. (**b**) Densitometry analysis of LC3 lipidation, as described prior; graph displays densitometry ratios of lipidated LC3 (LC3-II) to total LC3 (LC3-I + LC3-II). Representative blots from a single donor. (**c**) Cell lysates were subsequently run on a 4–12% Bis–Tris gel in MES buffer (Thermo Fisher) and proteins were transferred onto a 0.2 μm nitrocellulose membrane. ATG12 was detected by western blot and appears either as the ATG5-ATG12 complex (around 55 kDa) or as free ATG12. Results display representative blots from a single donor, original blot images are displayed in Supplementary Fig. S12. (**d**) AB-MAC and ABI-MAC were incubated overnight onto 15 mm coverslips in 12-well plates (6 × 10^5^ cells/well) and were either left untreated (Control) or treated with Chloroquine, Rapamycin, or a combination of both drugs. Cells were then fixed, permeabilized and stained with antibodies against LC3 (green) and LAMP2 (red); LC3-I displays a diffuse cytoplasmic staining while LC3-II exhibits a punctate pattern.

We next focused on LC3 proteins: these key markers of the autophagy process exist as free cytosolic forms in the absence of autophagy induction, hereafter referred to as LC3-I; once autophagy is induced LC3 become lipidated with phosphatidylethanolamine (LC3-II), tethering them to the nascent phagophore membrane^31–33^. Therefore, above-mentioned cell lysates were analyzed for LC3 lipidation levels (Fig. 4a)**.** Once more, to our surprise, we discovered that LC3 lipidation was not enhanced in ABI-MAC, despite the strong autophagy induction demonstrated previously through multiple assays. Actually, by analyzing LC3-II to total LC3 ratios across experimental samples (Fig. 4b), we found that control-treated AB-MAC and ABI-MAC exhibited similar lipidation levels, while mTOR-dependent autophagy induction through overnight rapamycin treatment greatly increased LC3 lipidation levels in both cell types. This result was confirmed by assessing ATG5-ATG12 complex formation, which promotes LC3 lipidation: there was no enrichment of this complex in IL-27-treated cells (Fig. 4c), nor could we see differences in LC3 lipidation levels or ATG5–ATG12 complex formation during monocyte-to-macrophage differentiation (Supplementary Fig. S7), both of which corroborate the LC3-independent character of IL-27–induced autophagy. Finally, we used immunostaining to further confirm these findings (Fig. 4d): after AB-MAC and ABI-MAC were treated with either chloroquine, rapamycin, or the chloroquine/rapamycin combination, cells were fixed and permeabilized, after which we assessed localization and expression levels of both LC3 and lysosome-associated membrane protein 2 (LAMP2), a lysosomal protein essential for autophagosome-lysosome fusion and subsequent autophagosome degradation. Corroborating our western blot results, we found that LC3 was mostly displaying diffuse cytoplasmic patterns in both untreated AB-MAC and ABI-MAC, the decrease of LC3 puncta in the latter confirming that despite a strong autophagy induction following IL-27 differentiation, those autophagic structures were indeed LC3-independent. Accumulation of those LC3 puncta in LAMP2-rich cytoplasmic regions could be seen after chloroquine addition to both cellular subsets, which blocked lysosomal-mediated autophagosome degradation, suggesting that basal canonical autophagy could, in fact, take place in both AB-MAC and ABI-MAC. Autophagy induction using rapamycin, either alone or in combination with chloroquine treatment, resulted in an even higher level of LC3 puncta formation, confirming that canonical mTOR-dependent autophagy was functional in both cell types. Taken together, these data confirmed that IL-27–induced autophagy in ABI-MAC was non-canonical and independent of both mTOR dephosphorylation and LC3 lipidation.

**Figure 5:**
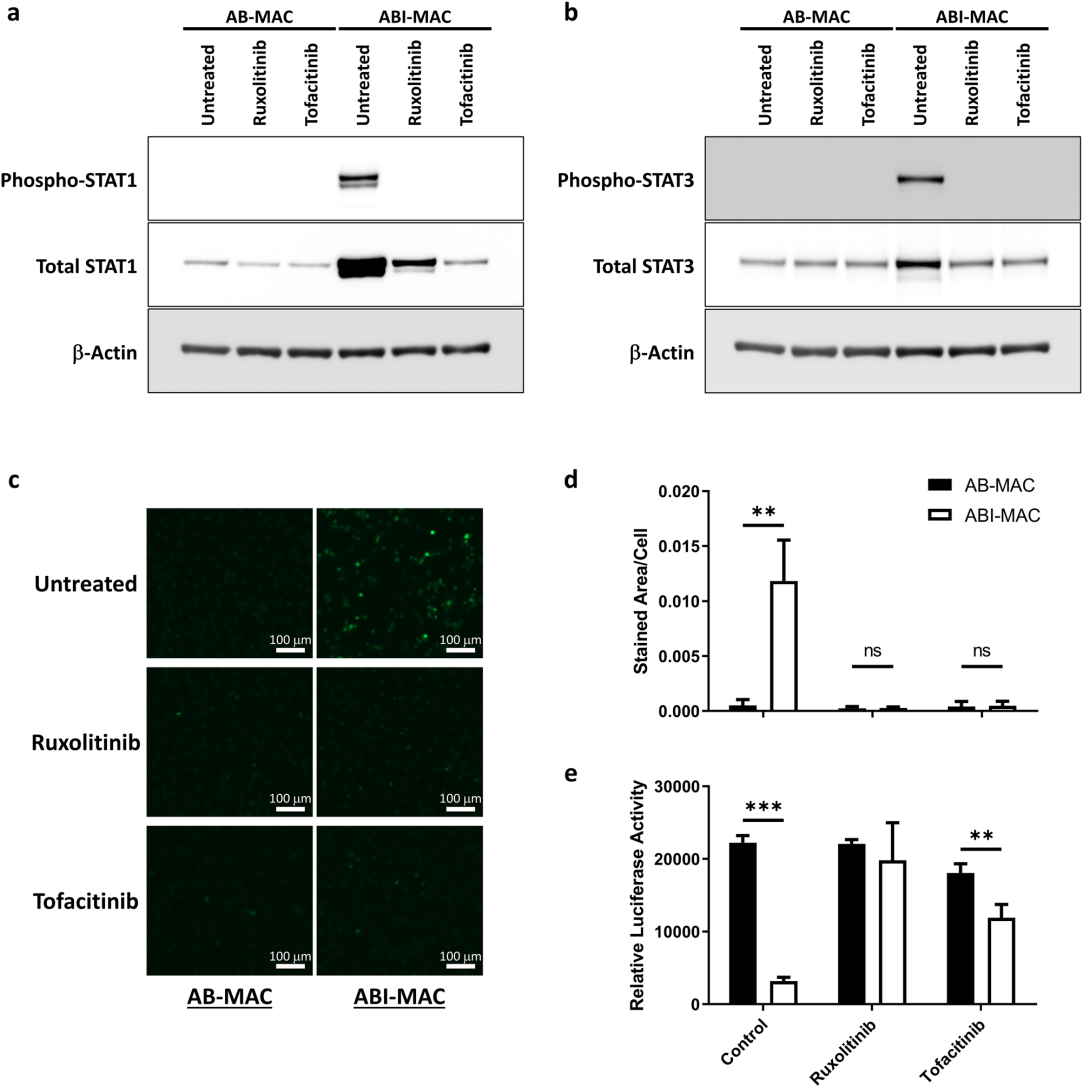
Impact of JAK inhibitors on autophagy induction and HIV inhibition. Monocytes were differentiated into AB-MAC and ABI-MAC either as described before or treated with Ruxolitinib or Tofacitinib. After differentiation, a first subset of cells was immediately lysed in RIPA buffer, proteins were run on 4–12% Bis–Tris gels in MOPS buffer then transferred onto 0.45 μm nitrocellulose membranes. Blots were probed for (**a**) phospho-STAT1, total STAT1 and β-Actin or (**b**) phospho-STAT3, total STAT3 and β-Actin as described in the “Materials and methods”. Results indicate representative blots from a single donor, original blot images are displayed in Supplementary Figs. S13 and S14. (**c**) Autophagosome staining was performed using the Cyto-ID reagent on a second subset of cells, and (**d**) images were quantified as described in the “Materials and methods”; displayed values represent mean and SD, and relevant Student’s *t*-test are indicated, **p < 0.01 (n = 4). (**e**) A third subset of cells was infected with HIVLuc-V and HIV infection assay was performed as described in the “Materials and methods”. Displayed values are mean relative luciferase activities from three independent donors; error bars denote standard deviations. Results of significant and relevant Student’s *t*-tests are indicated, **p < 0.01, and ***p < 0.001.

We previously reported that IL-27 mediates cellular signaling via Jak/STAT and TAK1/MAPK pathways in macrophages, and that the TAK1/MAPK pathway is involved in HIV resistance in IL27 + M-CSF-induced macrophages^5^. In an attempt to draw a correlation between autophagy induction and HIV inhibition, we induced ABI-MAC in the presence of two different JAK inhibitors, Ruxolitinib and Tofacitinib^34–36^: those treatments suppressed IL-27-mediated STAT1 and STAT3 activations (Fig. 5a,b, respectively) along with autophagy induction and HIV restriction (Fig. 5c–e, and Supplementary Fig. S8), suggesting that both autophagy and control of HIV infection in ABI-MAC are mediated via the JAK/STAT pathway. Thus, in the current study, we could not elucidate how autophagy induction in ABI-MAC is linked to anti-HIV effects.

## Discussion

IL-27 was originally identified as a T-cell stimulant, however it is currently known as an antiviral cytokine that inhibits HIV, HCV and HSV^7^. It is reported that IL-27 suppresses IFN-γ-mediated autophagy induction in *Mycobacterium tuberculosis*-infected macrophages, however a role for IL-27 on autophagy induction during monocyte-to-macrophages differentiation has not been investigated. In the current study, to our best knowledge we demonstrated for the first time that IL-27-mediated induction autophagy during monocyte differentiation depended on the stimulants with which macrophages were induced. Macrophages derived from monocytes show functional heterogeneity^37–39^ and can be polarized into M1-type (classically activated macrophage) and M2-type (alternatively activated macrophages); M2-type macrophages are induced by M-CSF and are divided into M2a, M2b, M2c, and M2d subcategories. Our data indicated that IL-27 induced autophagy in huAB but not in M-CSF-induced macrophages (M2-Macrophages), suggesting that the IL-27-indcued autophagy may be caused by stimuli / subtype dependent manner(s). It would be of interest to investigate the subtype dependency in the autophagy induction to define an autophagy-inducing spectrum in IL-27-treatment.

In our previous work, we have demonstrated that IL-27 promotes differentiation of monocytes into HIV-resistant macrophages in the presence of M-CSF^5^. Using a pseudotyped HIV, HIVLuc-V virus, in an infection assay, we found that the resistance was caused by suppressing HIV reverse transcription after cell entry and before integration^5^. In the current study, ABI-MAC demonstrated the same inhibitory effect when using HIVLUC-V infection. To precisely determine which step of the viral life cycle (from infection, reverse transcription and integration to viral transcription and translation of viral proteins) is suppressed in ABI-MAC, we may need to compare the copy number of proviral DNA or viral transcripts and the amounts of viral proteins between ABI-MAC and AB-MAC; nonetheless, similar to our previous work, our current data indicated that IL-27 can differentiate monocytes into HIV-resistant cells. Previously, we have also demonstrated that IL-27 triggers two independent cellular signaling: Jak/STAT and TAK1/MAPK pathways, and that the TAK1/MAPK pathway is involved in HIV resistance in IL27 + M-CSF-induced macrophages^5^. To define a role for TAK1/MAPK pathway in inducing autophagy in ABI-MAC, and to draw a link between anti-HIV effects and autophagy induction, we induced ABI-MAC in the presence of two JAK inhibitors, Ruxolitinib and Tofacitinib^34–36^: those treatments fully suppressed IL-27-mediated STAT1 and STAT3 activation (although Ruxolitinib only partially blocked total STAT1 overexpression in ABI-MAC) together with autophagy induction and HIV restriction, suggesting that the TAK/MAPK pathway has likely no impact on anti-HIV effects and autophagy induction seen in ABI-MAC. However, in the current study we could not elucidate how the autophagy induction in ABI-MAC links to anti-HIV effects, hence further studies are needed to define the correlation. Interestingly, we recently determined that bafilomycin A1 completely inhibits autophagosome formation in ABI-MAC, below basal autophagy levels seen in AB-MAC, while it had no significant effect on autophagosome formation in the latter (Supplementary Fig. S6). This could prove a powerful tool to decipher between the effects of IL-27 and autophagy vis-à-vis HIV inhibition in ABI-MAC.

The innate immune system regulates autophagy^11,40,41^, while pathogen infections can also modulate autophagy induction in host cells^42,43^; it has also been reported that autophagy induction correlates with HIV inhibition^17,18^; however, the degradation of HIV proteins by autophagy is repressed by an HIV protein, Nef^18^. As shown in Fig. 1, HIV-RED-V infection was inhibited in ABI-MAC, although HIV-RED-V expresses Nef^44,45^, suggesting that the Nef-mediated autophagy inhibition may not be functional in these cells. IL-27 suppresses not only HIV but also inhibits replication of herpes simplex virus^5^, hepatitis C virus^46^ and influenza virus^5^ in macrophages, T cells, and hepatocytes. It would be worth investigating whether IL-27-mediated autophagy induction is also involved in inhibiting these viruses in each cell type. As shown in Fig. 2a**,** IL-27 + M-CSF-induced macrophages did not display an increased autophagy activity; therefore, autophagy induction is triggered in a subtype dependent manner in macrophages. Intriguingly, we were able to transduce ABI-MAC with an LC3-RFP-expressing lentivirus (Supplementary Fig. S5), suggesting that those cells might still be permissible to some type of viral infection.

We showed here for the first time that IL-27 induces non-canonical autophagy in macrophages during differentiation. The microarray analysis demonstrated that the expression of myotubularin related protein 1, epsin 2, maternal embryonic leucine zipper kinase and cyclin dependent kinase 1 in ABI-MAC was modestly changed (2–3-fold downregulation) (Supplementary Table S1). These genes may regulate the mTOR/LC3 independent pathway of autophagy induction. Additionally, we were able to determine that ATG13 was differentially regulated in ABI-MAC: while mTOR-mediated induction of autophagy is fully functional in AB-MAC and ABI-MAC, and mTOR phosphorylation was similarly regulated upon rapamycin treatment in both cell types, mTORC1 phosphorylation of ATG13 was found to be inhibited in ABI-MAC. Future work should therefore focus on comparing ATG13, ULK1 and FIP200 modifications between AB-MAC and ABI-MAC. Further, molecular mechanisms of IL-27-mediated regulation of autophagy induction across different macrophage subtypes also need to be investigated, which may provide new insights for developing IL-27-mediated immunotherapy.

## Materials and methods

### Ethical approval statement

Approval for these studies, including all sample materials, was granted by the National Institute of Allergy and Infectious Diseases Institutional Review Board. All experimental procedures in these studies were approved by the National Cancer Institute at Frederick and Frederick National Laboratory for Cancer Research, and were performed in accordance with the relevant guidelines and regulations (protocol code number: 16–19, approval data: 6 January 2017). Informed consent was obtained from all subjects involved in the study and all participants provided written consent prior to blood being drawn.

### Induction of macrophages

Peripheral blood mononuclear cells (PBMCs) were isolated from healthy donor’s apheresis (Blood Bank, National Institute of Health, Bethesda, MD) using lymphocyte separation medium (MPbio, Solon, OH, USA) as previously described^5^. CD14+ monocytes were isolated from PBMCs using MACS CD14 MicroBeads (Miltenyi Biotec, Auburn, CA, USA). Monocytes were differentiated into macrophages in D10 medium [D-MEM medium (Thermo Fisher Scientific, Waltham, MA) supplemented with 10% (v/v) heat-inactivated Fetal Bovine Serum (FBS) (Hyclone, GE-Health Care, Chicago, IL), 10 mM 4-(2-hydroxyethyl)-1-piperazineethanesulfonic acid (HEPES), pH 7.4 (Quality Biology, Gaithersburg, MD) and 5 µg/ml of Gentamycin (Thermo Fisher)] with 10% (v/v) pooled human AB serum (Gemini Bio, West Sacramento, CA) in the presence or absence of 100 ng/ml of IL-27 (R&D systems, Minneapolis, MN) for 7 days. Macrophage-colony stimulatory factor (M-CSF)-induced MDMs were prepared using 25 ng/ml of M-CSF (R&D systems) with or without 100 ng/ml of IL-27 in macrophage-serum free medium (Thermo Fisher Scientific) supplemented with 10 mM HEPES, pH7.4 and 5 µg/ml of Gentamycin for 7 days. For both cell type, half the culture medium was replaced on the 4th day with fresh corresponding medium with required supplements. Differentiated macrophages were maintained in D10 medium as previously described^5^. HEK293T cells (ATCC, Manassas, VA, USA) were maintained in D10 medium as previously described^5^.

### Recombinant HIV-1 viruses

VSV-G-pseudotyped HIV-luciferase virus (HIVLuc-V) and red fluorescent protein (DsRed)-tagged HIV (HIV-RED-V) were prepared by co-transfecting HEK293T cells with pNL4-3ΔEnv-Luc^47,48^ or pNLDsRED-ΔEnv^44,45^ and pLTR-VSVG5 using the TransIT-293 transfection reagent (Mirus, Madison, WI) as previously described^5^. The pNLDsRED-ΔEnv construct was created from pNLDsRED^44^ by inducing a stop codon in env gene^45^. Virus-containing supernatants of HEK293T cell culture were harvested 48 h after transfection and filtered with 0.45-μm Steriflip Filter Units (MiliporeSigma, Louis, MO, USA). Virus particles were pelleted by ultracentrifugation at 100,000×*g* for 2 h, at 4 °C on a 20% Sucrose in 150 mM NaCl-HEPES, pH7.4 buffer and resuspended in D10 medium. Virus concentration in the suspension was quantified by a p24 antigen capture kit (PerkinElmer, Waltham, MA, USA) and stored at -80 °C until use.

### HIV infection assay

HIV-1 Infection assay was performed using the pseudotyped HIVLuc-V or HIV-RED-V as previously described^5^. Both recombinant viruses express VSV-G envelope protein on the virus surface instead of HIV Env protein. The viruses can enter macrophages without using HIV receptor (CD4 and chemokine receptors) and complete only a single round of infection. As such, the system allowed us to focus on HIV-1 infection at a post-entry level. AB-MAC or ABI-MAC were seeded in 96 well plate (50 × 10^3^ cells/well), cultured for 4 h at 37 °C and then infected with 100 mL of 100 ng p24/ml of HIVLuc-V or HIV-RED-V for 2 h at 37 °C. Infected cells were washed twice with warm D10 medium and then cultured for 24 h at 37 °C. To measure luciferase activity, cells were lysed in 1× Passive Lysis Buffer (Promega, Madison, Wisconsin, USA) and luciferase activity was measured using the Luciferase Assay System (Promega). We also retrieved protein concentrations from cell lysates as a measure of cell number using the BCA protein assay kit (Thermo Fisher).

### HIV infection and autophagy detection

To determine a correlation between HIV infection and autophagy induction in macrophages, AB-MAC and ABI-MAC were infected with the pseudotyped HIV-RED-V and counterstained with Cyto-ID reagent (Enzo Life Sciences, Farmingdale, NY). 2 × 3 composite images were taken on a Zeiss Axio Observer.Z1-motorized microscope (Zeiss, Oberkochen, Germany) at a 10× magnification; image analysis was performed using the FiJi software^49^. Image segmentation was carried out for each composite image based on the DIC channel in RGB mode, as follows: contrast was first increased using the ‘Enhance Contrast’ tool (0.0% saturation, histogram equalization turned on). The ‘Find Edges’ tool was used to outline cells, and images were sequentially applied ‘Minimum’ (radius = 0 px) and ‘Maximum’ (radius = 5 px) filters to further enhance cell separation. Resulting images were then smoothed, converted to 8 bit and binary masks were created following background subtraction, after which we used the ‘Gray Morphology’ tool to (1) close holes, (2) dilate then (3) erode structures. Finally, the ‘Analyze Particles’ tool following watershed processing was run and resulting region of interests (ROIs) were added to the manager; those coordinates were used to retrieve mean fluorescence intensities from both green (Cyto-ID) and red (HIV-RED-V) channels for each individual ROI. To define the correlation between HIV infection and autophagy, scatter plot analysis was performed between HIV-RED-V vs Cyto-ID for each cell type.

### Cell preparation for autophagy staining and quantification

AB-MAC, ABI-MAC, M-CSF-induced macrophages or M-CSF with IL-27-induced macrophages were plated at 50 × 10^3^ cells/well in 96-well plates and incubated for 4 h at 37 °C. Cells were then incubated overnight in D10 medium with or without 10 μM chloroquine. As a positive control for autophagy induction, cells were treated with a combination of chloroquine and rapamycin (500 nM). Autophagosome staining was performed using the Cyto-ID reagent and Hoechst 33342 as a counterstain, as per manufacturer instructions and described previously^50^. Briefly, culture medium was removed and replaced with 5% FBS-supplemented PBS containing both stains. Cells were then incubated at 37 °C for 30 min prior to imaging. 2 × 2 composite images were taken on a Zeiss Axio Observer.Z1 motorized microscope at a 10× magnification; image analysis was performed using the FiJi software^49^. For each channel, a background threshold was set up in order to create 8-bit masks. For the green channel, the total stained area was retrieved as a measure of autophagosome staining, while a particle count following watershed processing of the blue channel gave the corresponding cell number. Results therefore display the average stained area per cell for each experimental condition.

### LC3 and LAMP2 immunostaining

Differentiated AB-MAC and ABI-MAC were plated on round 15 mm #1 glass coverslips (VWR) in 12-well plates (1.5 × 10^6^ cells/well) and incubated for 4 h at 37 °C. Cells were then incubated overnight in D10 medium with or without 10 μM chloroquine, 500 nM rapamycin, or a combination of chloroquine and rapamycin. Culture medium was then removed, and cells were fixed with ice-cold MetOH for 15 min at − 20 °C; fixative was subsequently removed, and cells were washed four times in PBS (Quality Biological). Cells were then permeabilized using 0.3% Triton (Calbiochem) in PBS for 10 min at room temperature, then washed four times in PBS, after which cells were blocked using the BlockAid™ blocking solution (ThermoFisher Scientific) for 1 h at room temperature. Coverslips were then recovered from plates and placed upside-down on a 100 μl drop of antibody-containing BlockAid™ and incubated in a humid chamber overnight at 4 °C in the dark. Coverslips were then washed four times in PBS, incubated with respective secondary antibodies (ThermoFisher Scientific) for 2 h at room temperature, washed four times in PBS, than mounted on glass slides in a drop of ProLong™ Diamond antifade mountant with DAPI (ThermoFisher Scientific). Imaging was carried out on a Zeiss Axio Observer.Z1 motorized microscope using a Plan-Apochromat 63x/1.40 objective and the LSM800 confocal module. Anti-LC3 (1:200, Cat# 2775) was obtained from Cell Signaling Technology (Danvers, MA, USA), anti-LAMP2 (1:100, Cat# ab25631) was purchased from Abcam (Cambridge, MA, USA).

### Microarray analysis

AB-MAC and ABI-MAC were seeded in 6 well plate (1.5 × 10^6^ cells/well) and cultured for 16 h at 37 °C. Prior to extracting RNA, cells were washed with cold PBS and then lysed in Qiazol (Qiagen, Germantown, MD, USA); total RNA was extracted using the RNeasy Micro Kit (Qiagen, Germantown, MD, USA). RNA quantification and quality were assessed using standard procedures by Nanodrop (Thermo Fisher Scientific) and Bioanalyzer (Agilent, Santa Clara, CA 95051). Gene expression profiles in AB-MAC and ABI-MAC were compared using the Affymetrix Human Transcriptome 2.0 Array (Thermo Fisher) as previously described^5^. Statistical data analysis for six arrays (AB and ABI-MAC from three independent donors) focusing on autophagy-related genes (ATGs) was done using Partek Pro (Partek, St. Louis, MO, USA). ATGs were collected from the following databases: Human Autophagy Moderator database (http://hamdb.scbdd.com), Human Autophagy Database (http://autophagy.lu), and Autophagy Database (http://www.tanpaku.org/autophagy).

### Transmission electron microscopy

AB-MAC and ABI-MAC were seeded in 6-well plate (1 × 10^6^ cells/well) for 16 h at 37 °C, and then washed with warm phosphate-buffered saline. Cells were fixed using 2% glutaraldehyde in cacodylate buffer (0.1 M sodium cacodylate trihydrate [MiliporeSigma], pH 7.0) for one hour at room temperature (in the dark), then overnight at 4 °C, and processed for electron microscopy (EM) analysis, as previously described^51^.

### Western blotting

Cell samples for Western blot analysis were prepared using radioimmunoprecipitation assay buffer (Boston biology, Boston, MA, USA) supplemented with both mammalian proteinase inhibitor cocktail (MiliporeSigma) and the Halt phosphatase inhibitor cocktail (Thermo Fisher). Total protein concentration in samples was quantified using the BCA protein assay kit (Thermo Fisher) and western blot analysis was conducted using 15 μg of total cell lysate. Protein samples were separated in 4–12% Bis–Tris gels (Thermo Fisher) with MOPS running buffer (Thermo Fisher) for STAT1, STAT3 and ATG13 phosphorylation analysis, 4–12% Bis–Tris gels (Thermo Fisher) with MES running buffer (Thermo Fisher) for LC3 lipidation and ATG5-ATG12 complex formation analysis, or 3–8% Tris–Acetate gel (Thermo Fisher) with Tris–acetate SDS-running butter (Thermo Fisher) for mTOR phosphorylation analysis. Western blot analysis was conducted as previously described^5^. Anti-phospho-mTOR (Ser2448) (Cat# 2971), anti-mTOR (Cat# 2972), anti-phospho-STAT3 (Tyr705) (Cat# 9145), anti-STAT3 (Cat# 12640), anti-phospho-STAT1 (Tyr701) (Cat# 9167), anti-STAT1 (Cat# 9176) and anti-phospho-ATG13 (Ser355) (Cat# 46329) were obtained from Cell Signaling Technology (Danvers, MA, USA), anti-LC3 antibody (Cat# ab48394), anti-ATG13 (Cat# ab201467) and anti-ATG12 (Cat# ab155589) were purchased from Abcam (Cambridge, MA, USA). Horseradish peroxidase-conjugated anti-Rabbit Ig and anti-mouse Ig antibodies (Cat#:NA931V and NA934V) were obtained from Thermo Fisher. Signal was detected using Amersham ECL Prime Western Blotting Detection Reagent (Cytiva, Global Life Sciences Solutions USA, Marlborough, MA, USA) and the ChemiDoc MP Imaging System (Bio-Rad, Hercules, CA, USA) as previously described^5^.

### Statistical analysis

Results were representative of at least three independent experiments. The values are expressed as means ± SD or ± SE, as indicated. Statistical significance was determined by two-tail, unpaired *t*-test using Prism 8.

## Supplementary Information

Supplementary Information.
