# Supplementary material for: Interleukin-27 promotes autophagy in human serum-induced primary macrophages via an mTOR- and LC3-independent pathway
Source: Sci Rep. 2021 Jul 21;11:14898. doi: 10.1038/s41598-021-94061-3 (PMC8295388; doi:10.1038/s41598-021-94061-3)
Supplement: Supplementary file 1 — Supplementary Information. [file 41598_2021_94061_MOESM1_ESM.pdf]

Supplementary Information
(Fig. S1-S16 and S. Table 1)

Interleukin-27 promotes autophagy in human serum-induced primary macrophages via an mTOR- and LC3-independent pathway

Sylvain Laverdure, Ziqiu Wang, Jun Yang, Takuya Yamamoto,

Tima Thomas, Toyotaka Sato, Kunio Nagashima,

and Tomozumi Imamichi

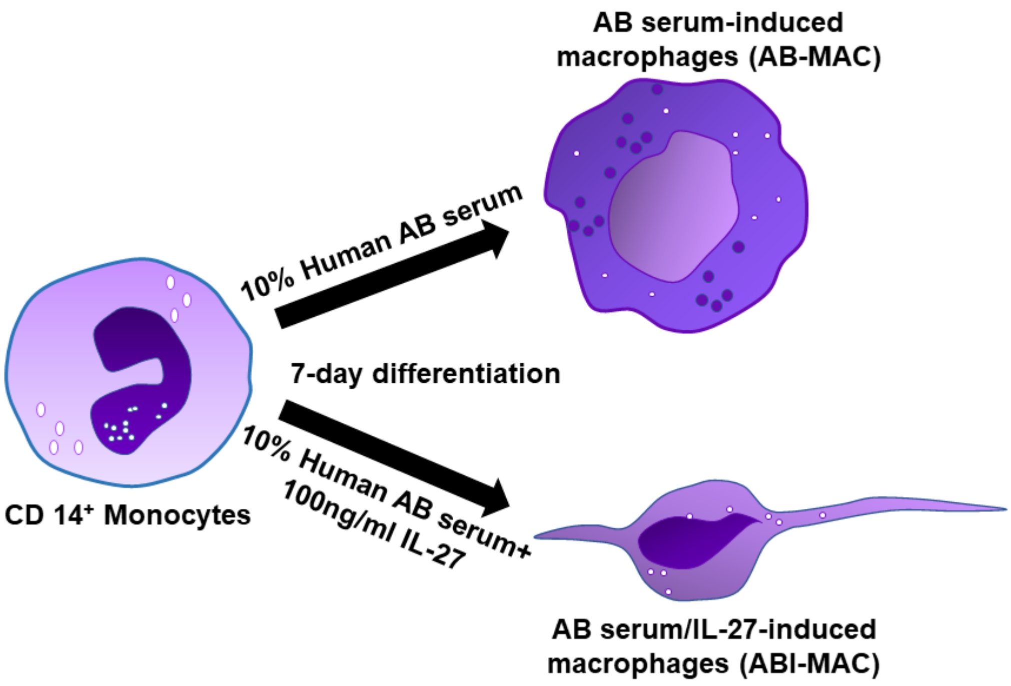

Supplementary Figure S1: A diagram of the preparation of AB-MAC and ABI-MAC. Freshly isolated CD14+ monocytes were differentiated into macrophages for 7-days using either 10% human AB serum in D10 media, generating AB-MAC, or a combination of 10% AB serum and IL-27 (100ng/mL) in D10 media, thereby creating ABI-MAC. A half of culture media was replaced with fresh D10 containing 10% huAB alone or huAB with IL-27. Differentiated macrophages were maintained D10.

a

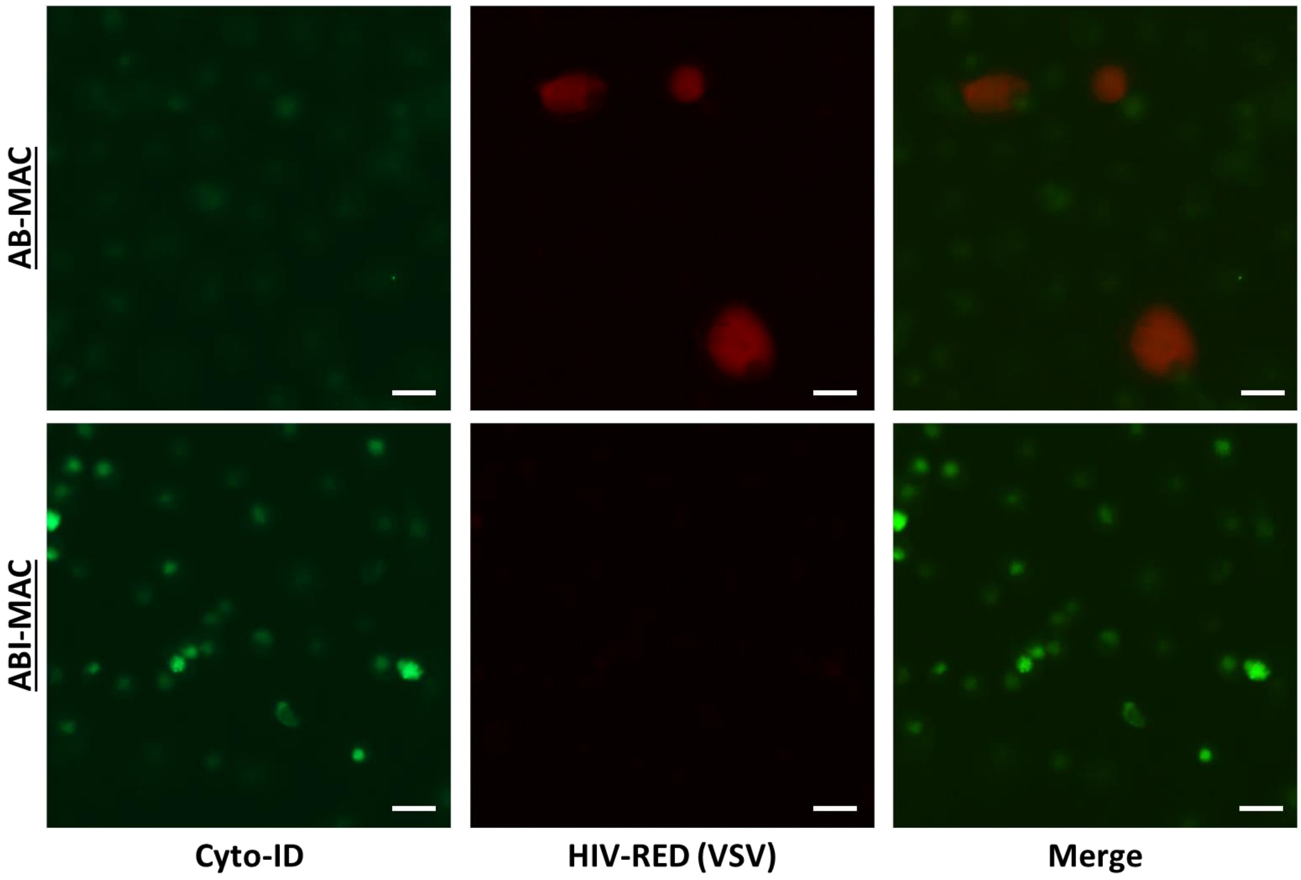

b

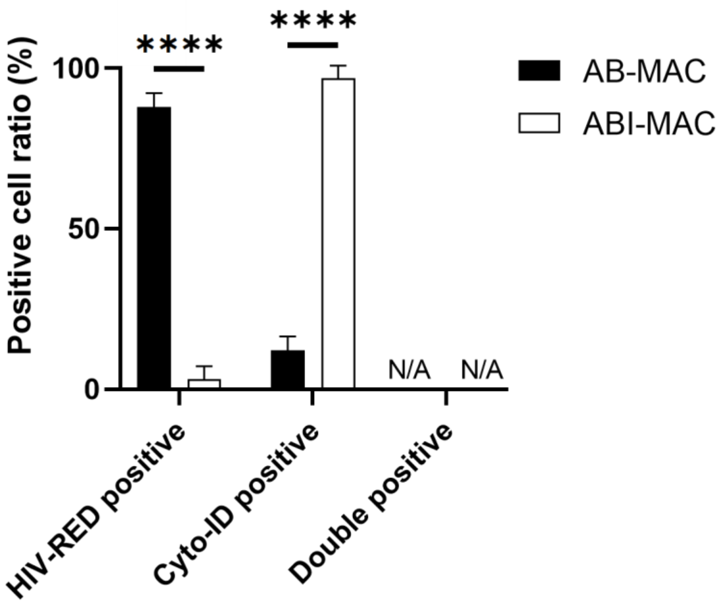

Supplementary Figure S2: HIV-RED and autophagy induction are mutually exclusive. AB-MAC and ABI-MAC were differentiated for 7 days, then infected with HIV-RED-V; 24 hours post-infection, cells were stained using the Cyto-ID staining kit. (a) HIV-infected cells are red, autophagic compartments appear green. Scale bar: 25μm. (b) DIC-based image segmentation was performed, allowing for single-cell level analysis of HIV-RED and Cyto-ID staining from AB-MAC and ABI-MAC. Bar graph shows distribution of positive cells among HIV-RED positive, Cyto-ID positive and double positive categories. Values represent mean and SD from 3 independent experiments. Results of relevant Student's *t*-test are indicated, **** representing $p<0.0001$.

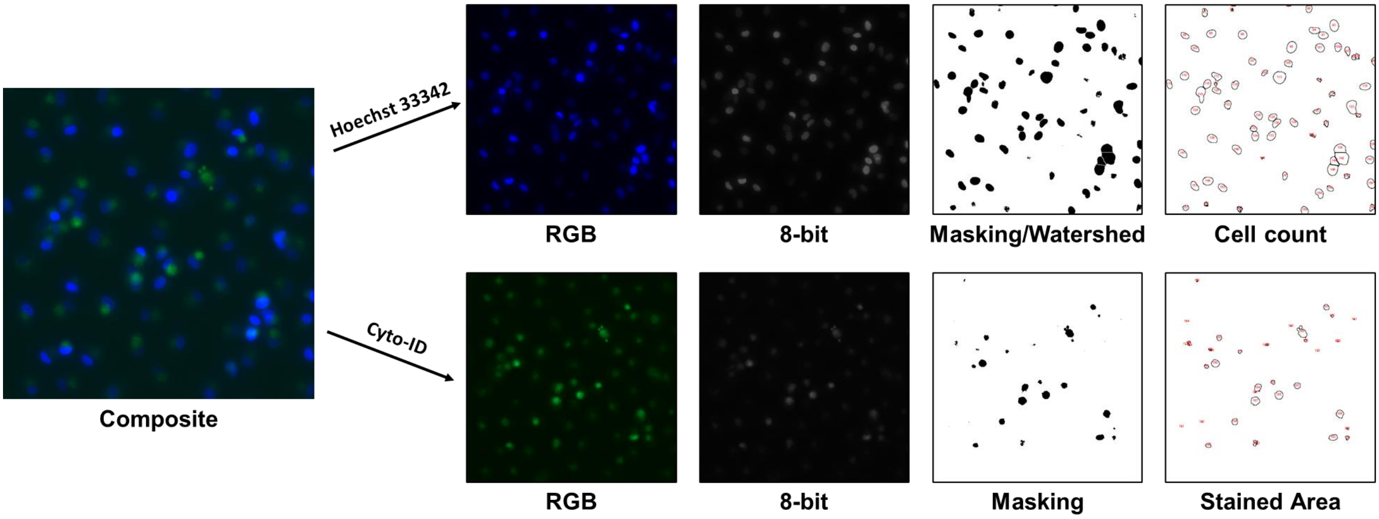

Supplementary Figure S3: A protocol for single-cell image analysis of autophagy induction.

Macrophages were incubated overnight in D10 medium with or without chloroquine (10 μM) or a combination of chloroquine and rapamycin (500 nM). Autophagosome staining was then performed using the Cyto-ID reagent, and Hoechst 33342 as a counterstain. 2x2 composite images were taken on a Zeiss AxioObserver motorized microscope at a 10x magnification. Cell image analysis was performed using the FiJi software. For each channel, a background threshold was set up in order to create binary masks. For the green channel, the total stained area was retrieved as a measure of autophagosome staining, while a particle count following watershed processing of the blue channel gave the corresponding cell number. Autophagy intensity was defined as the mean green area per cell.

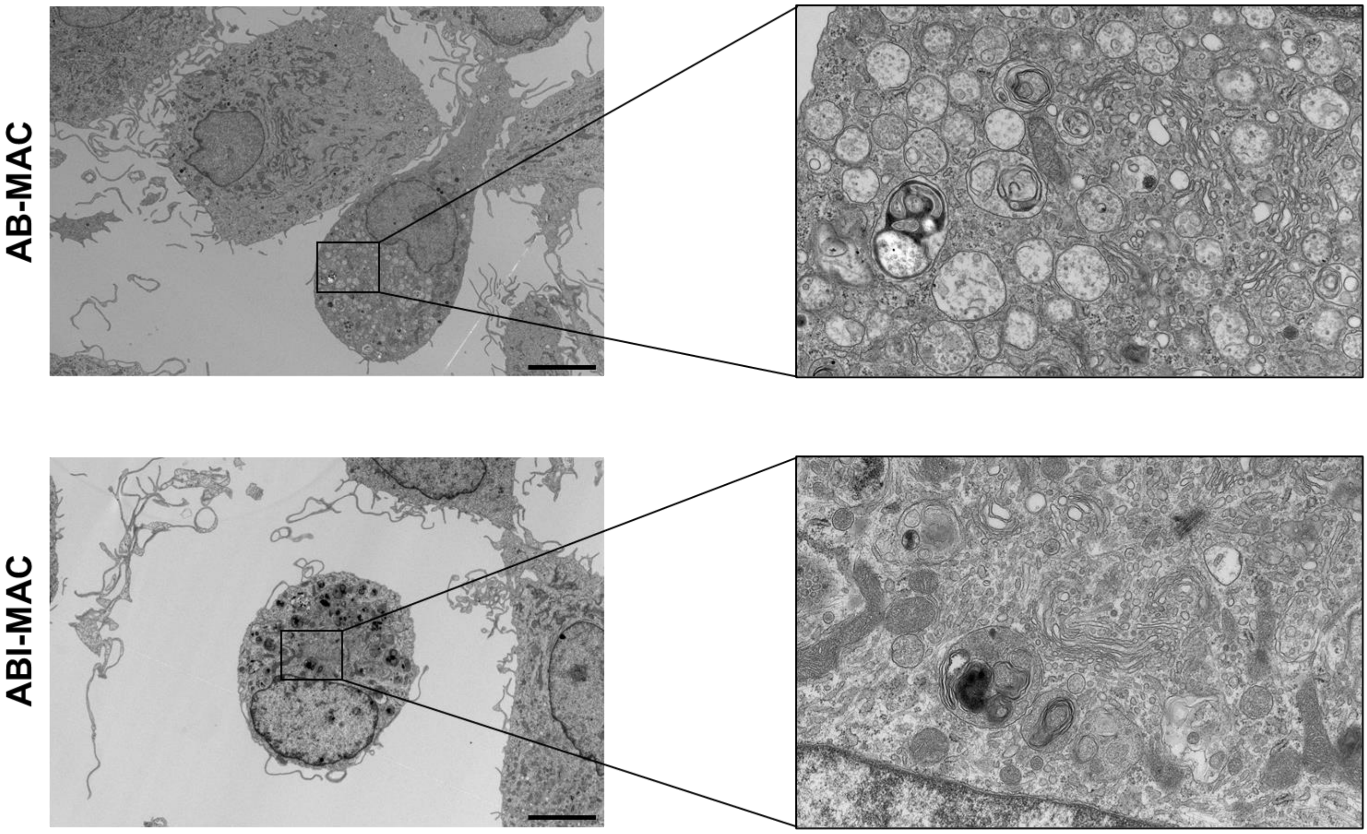

Supplementary Figure S4: Autophagosome enrichment following chloroquine treatment.

AB-MAC and ABI-MAC were seeded in 6-well plates and treated with chloroquine (10 μM) for 16 h at 37°C, and then washed with warmed phosphate-buffered saline prior to fixation and embedding. TEM analysis revealed an enrichment in autophagosomes for both cell types, when compared to control-treated cells. Boxed ROIs are magnified for organelle analysis. Scale bar: 6μm.

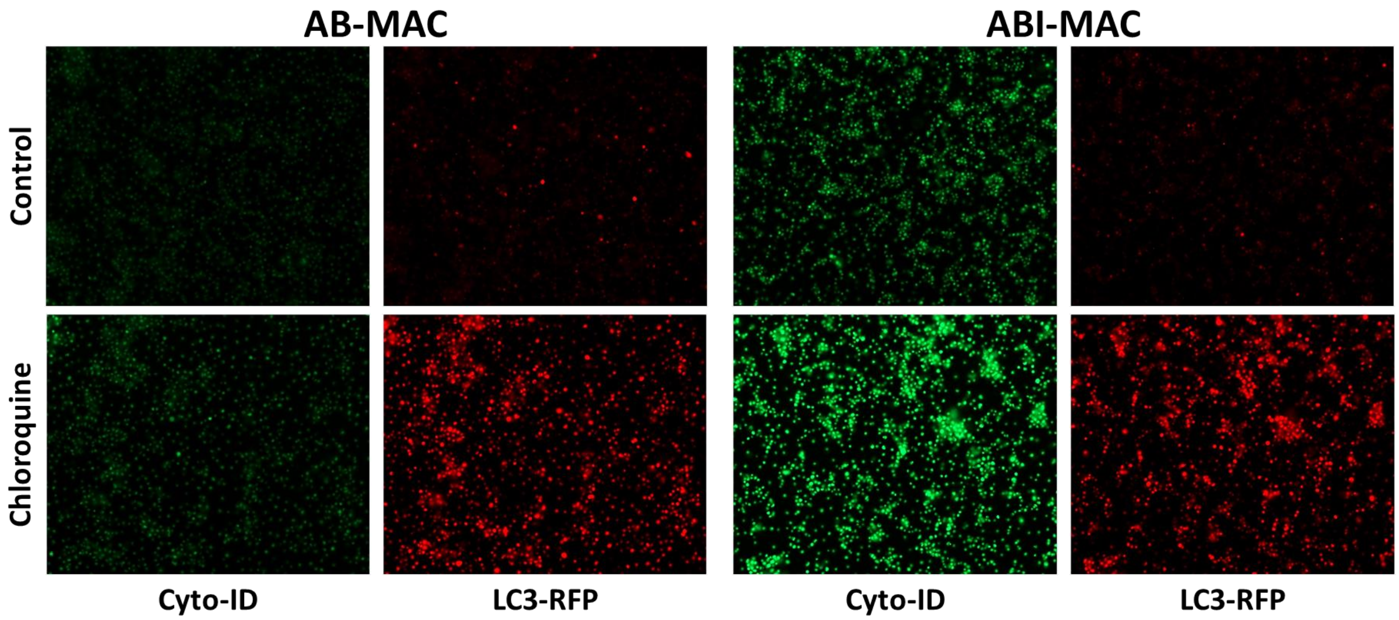

Supplementary Figure S5: Transduction of AB-MAC and ABI-MAC with the LentiBrite RFP-LC3 Lentiviral Biosensor.
AB-MAC and ABI-MAC were differentiated for 7 days, harvested, plated in 96-w plates and infected with the lentiviral vector at MOI=20 (555ng of p24/mL). 24 hours post-infection, cells were treated with chloroquine (10 μM) or left untreated. 48 hours post-infection, cells were stained using the Cyto-ID reagent and imaging was carried out immediately. Autophagic compartments are stained green, LC3-positive compartments appear red. LC3-RFP levels were similar for both cell types, both in control and chloroquine-treated cells, while the Cyto-ID staining still showed enrichment of the autophagic compartment in IL-27-treated cells.

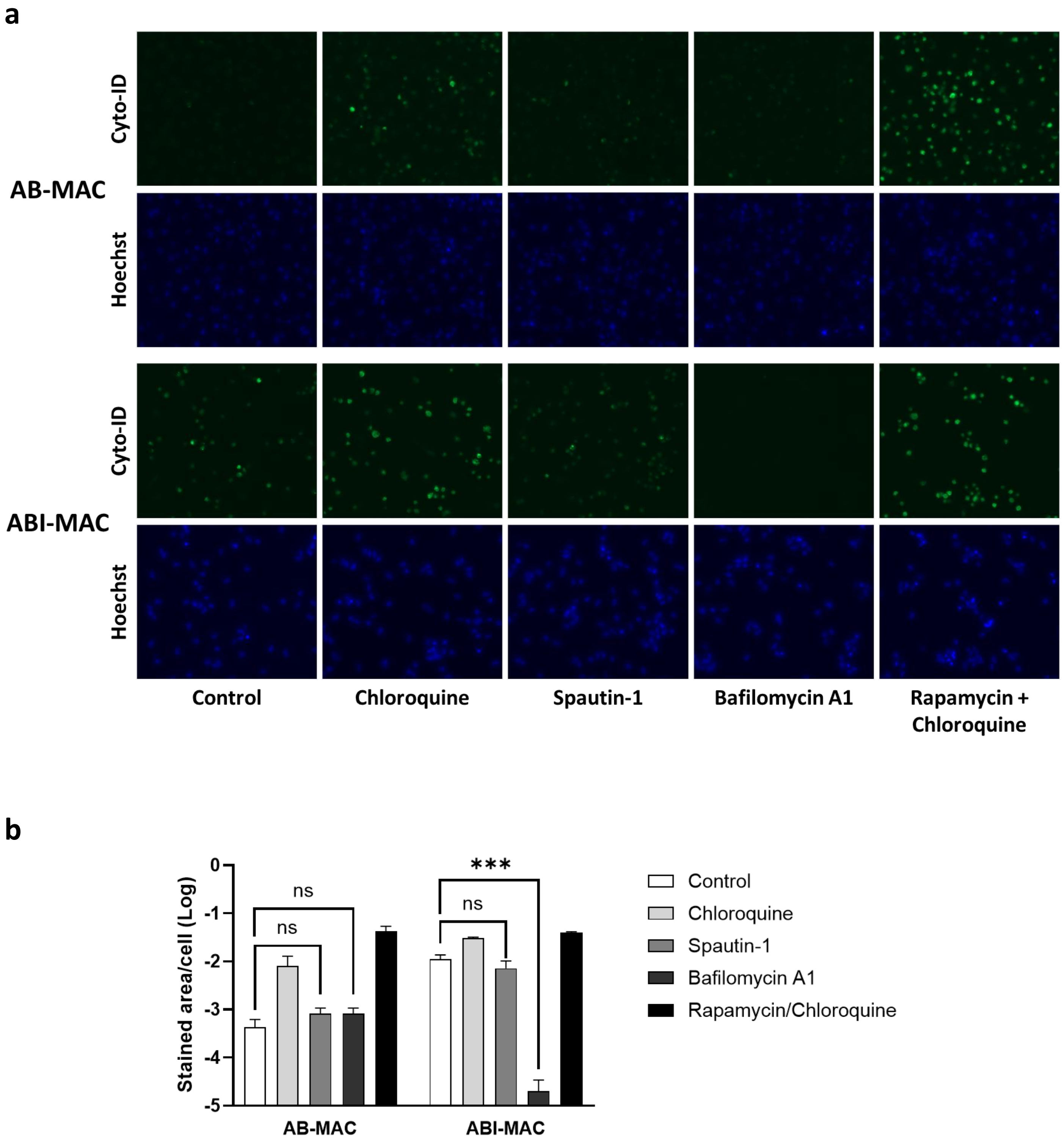

Supplementary Figure S6: Bafilomycin A1 inhibits autophagy induction in ABI-MAC. Monocytes were differentiated into AB-MAC and ABI-MAC for 7 days. After differentiation, cells were plated in 96-w plates and either left untreated (control) or treated overnight with chloroquine (10μM), Spautin-1 (15μM), Bafilomycin A1 (100nM), or a combination of Chloroquine and Rapamycin (500nM) as a positive control. After incubation, (a) autophagosome staining was performed using the Cyto-ID reagent, and Hoechst 33342 as a counterstain. (b) Autophagy induction was measured as described before, bar graph represents average stained area per cell for each experimental condition, +/- SD. Relevant Student's *t*-tests results are indicated, ***= <0.0001.

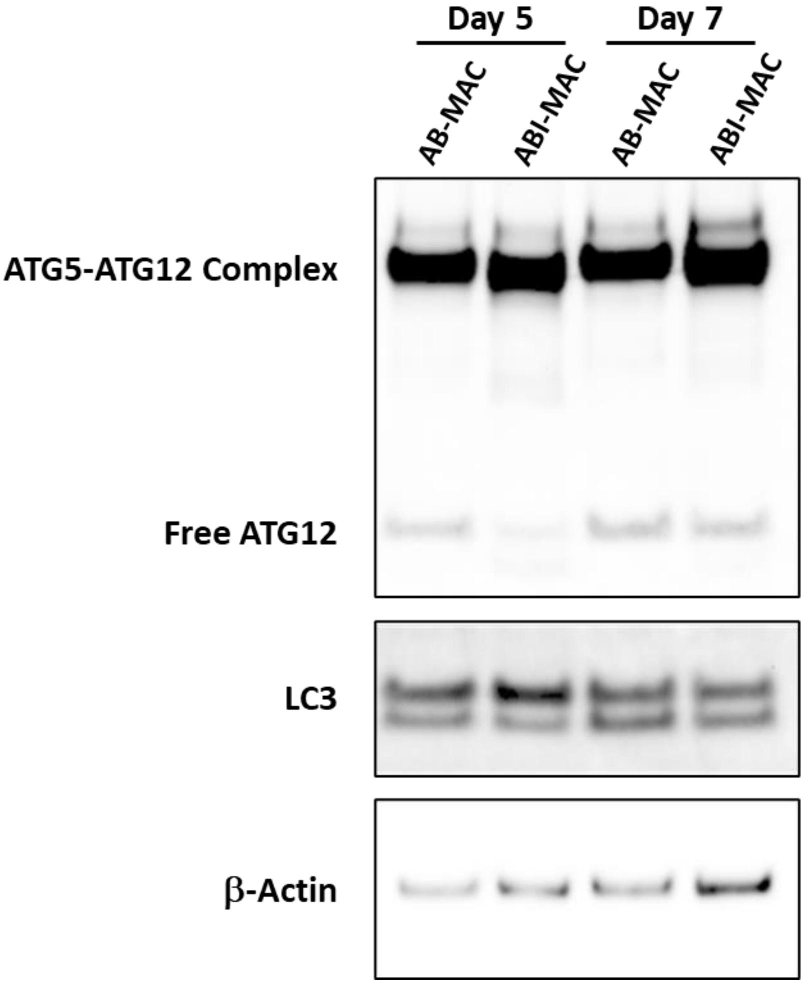

Supplementary Figure S7: ATG5-ATG12 complex formation and LC3 lipidation during differentiation.

Monocytes were differentiated into AB-MAC and ABI-MAC, cells were collected after 5 and 7 days of differentiation, lysed in RIPA, then samples were analyzed using western blot to assess LC3 lipidation and ATG5-ATG12 complex formation during differentiation. Original blot images are displayed in Supplementary Figure S15.

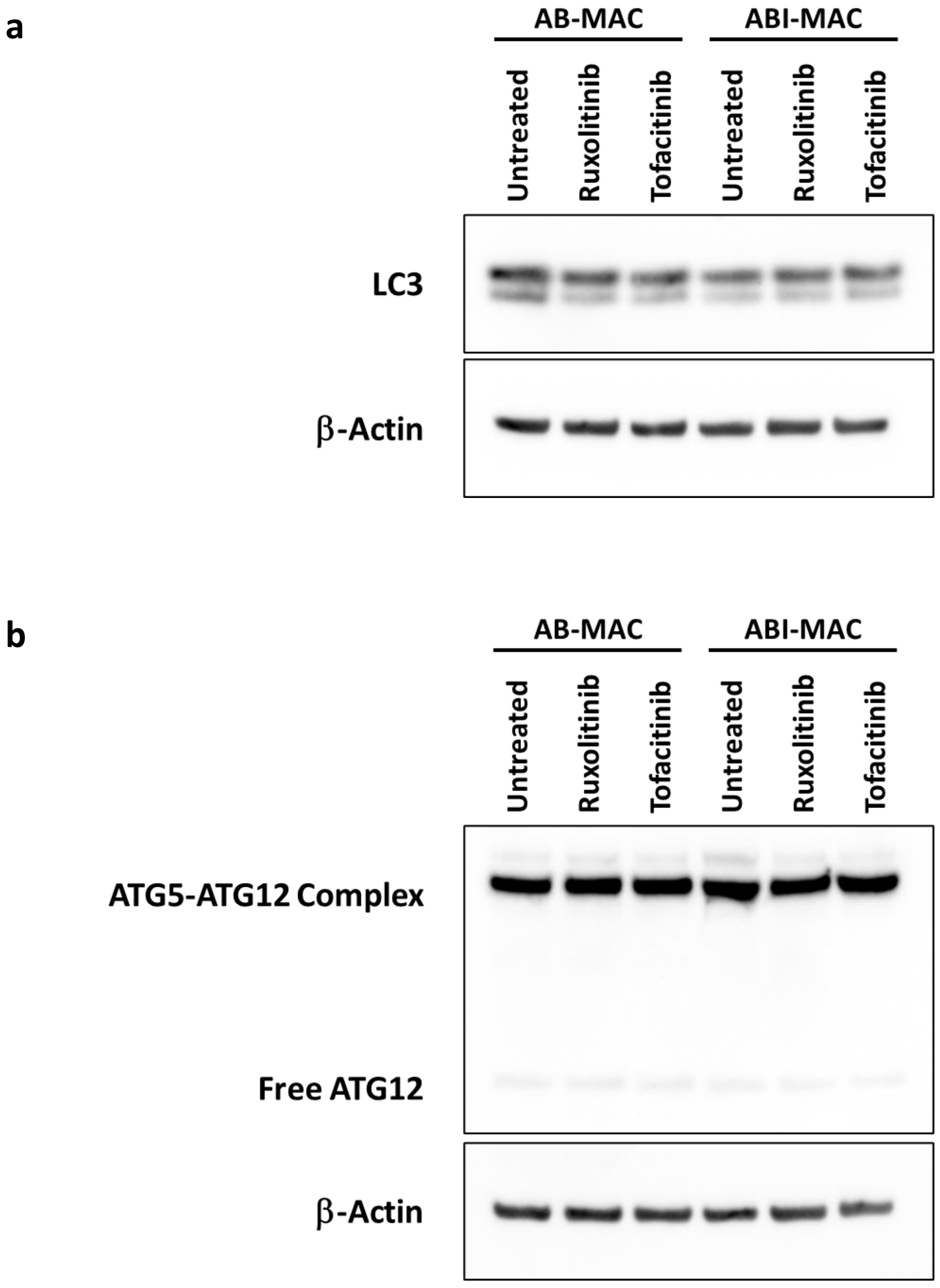

Supplementary Figure S8: ATG5-ATG12 complex formation and LC3 lipidation following treatment with JAK inhibitors.
Monocytes were differentiated into AB-MAC and ABI-MAC, either untreated or treated with Ruxolitinib or Tofacitinib. After differentiation, cells were lysed in RIPA, then samples were analyzed using western blot to assess (a) LC3 lipidation and (b) ATG5-ATG12 complex formation after treatment with either inhibitor. Original blot images are displayed in Supplementary Figure S16.

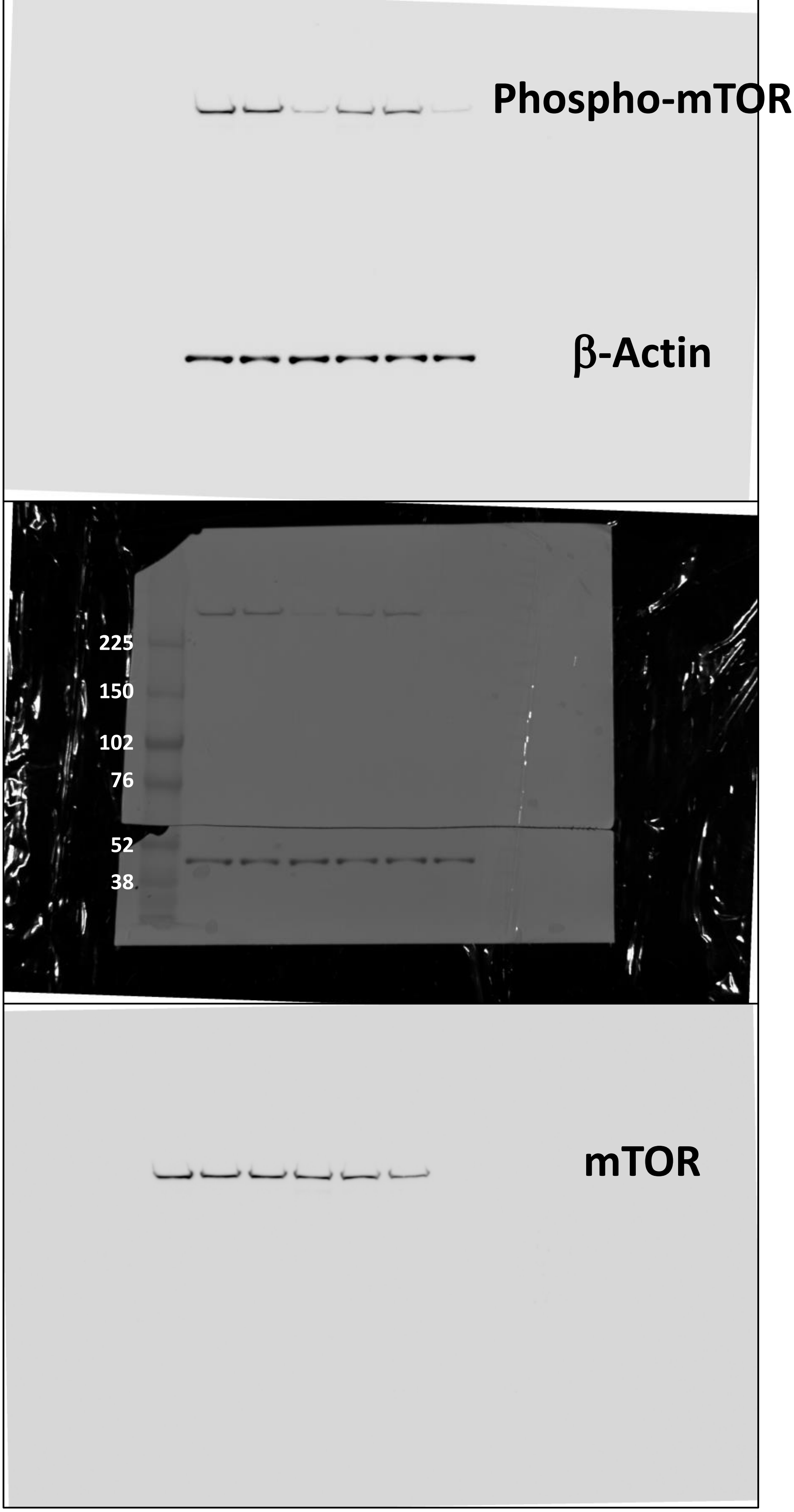

Supplementary Figure S9: Original blots for Figure 3a
This figure includes original blots as well as a composite overlay image displaying a molecular weight marker (relevant kDa values are indicated).

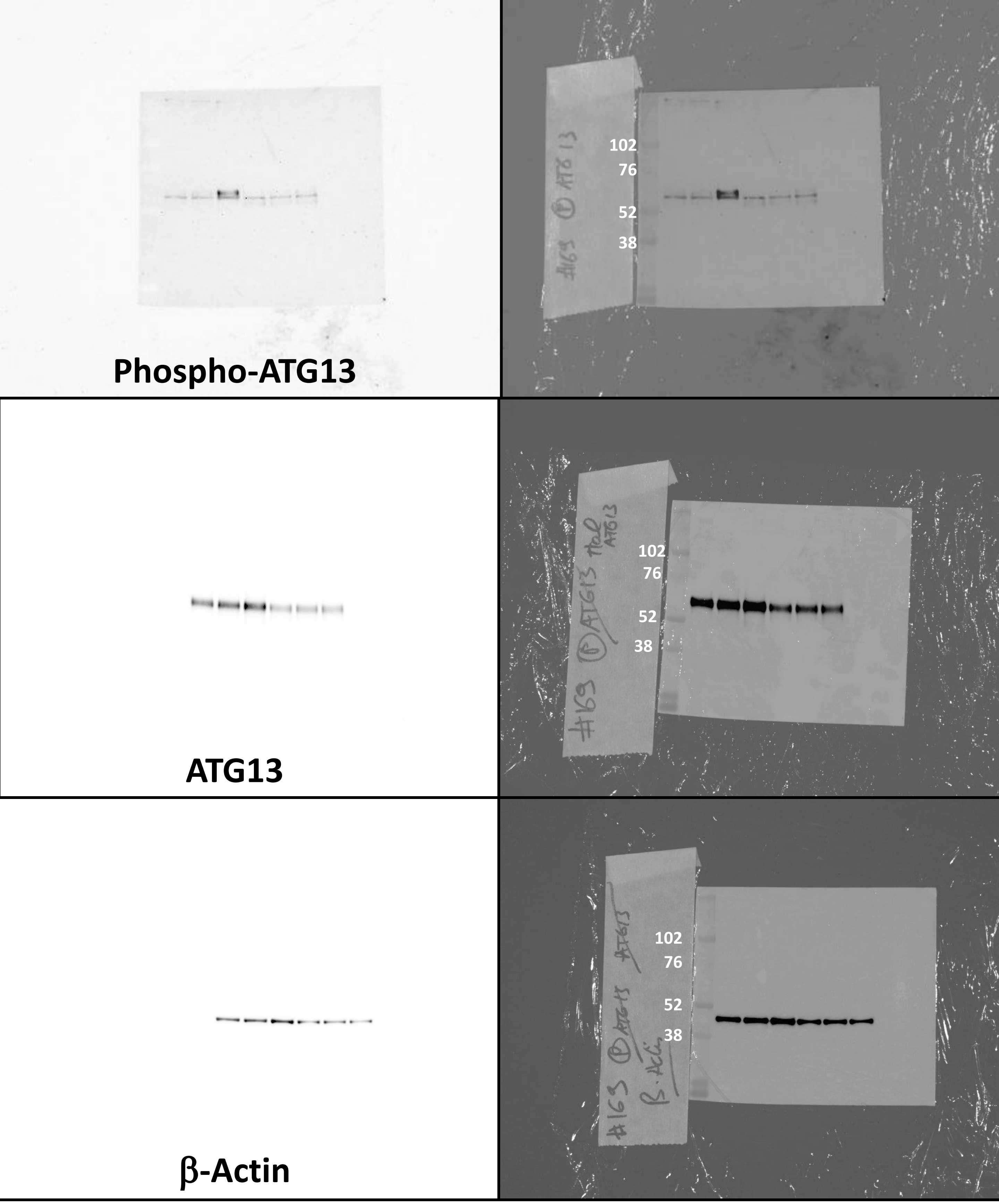

Supplementary Figure S10: Original blots for Figure 3c
This figure includes original blots as well as composite overlay images displaying molecular weight markers (relevant kDa values are indicated).

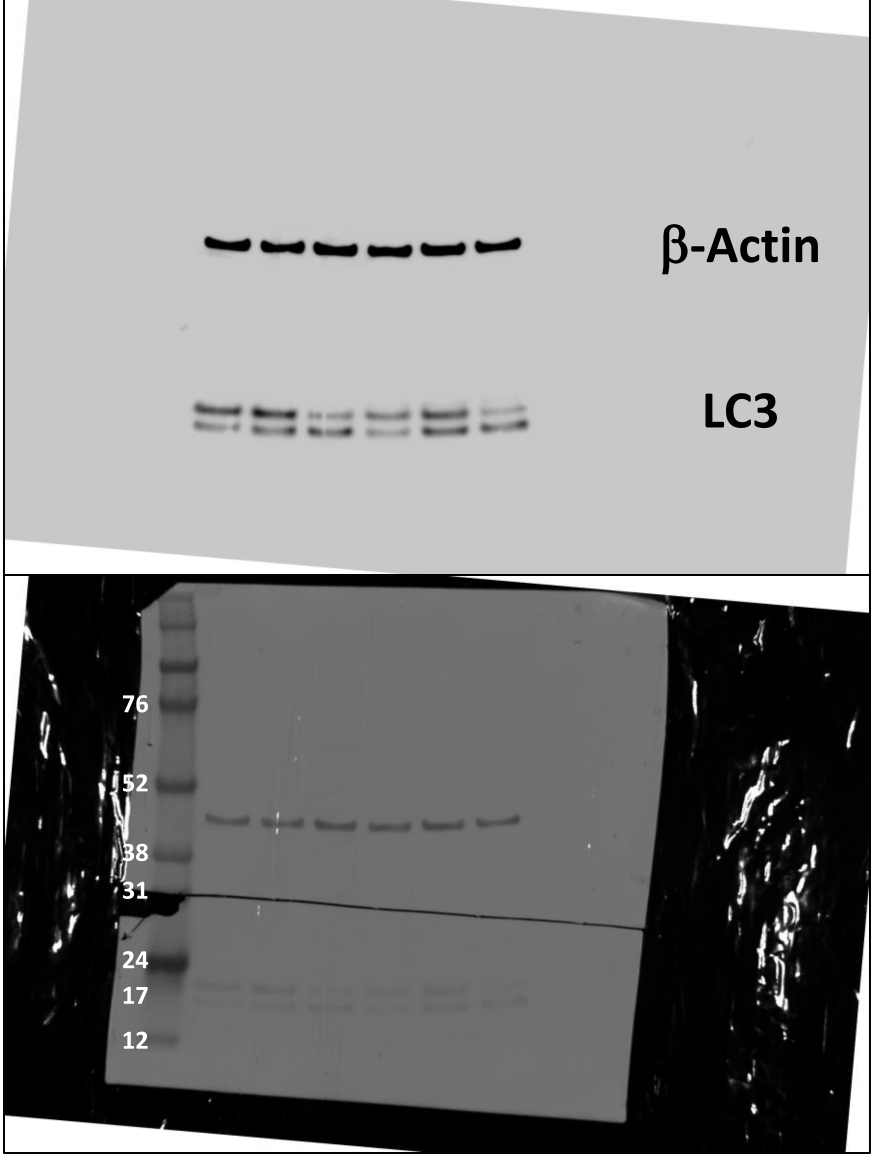

Supplementary Figure S11: Original blots for Figure 4a

This figure includes original blots as well as a composite overlay image displaying a molecular weight marker (relevant kDa values are indicated).

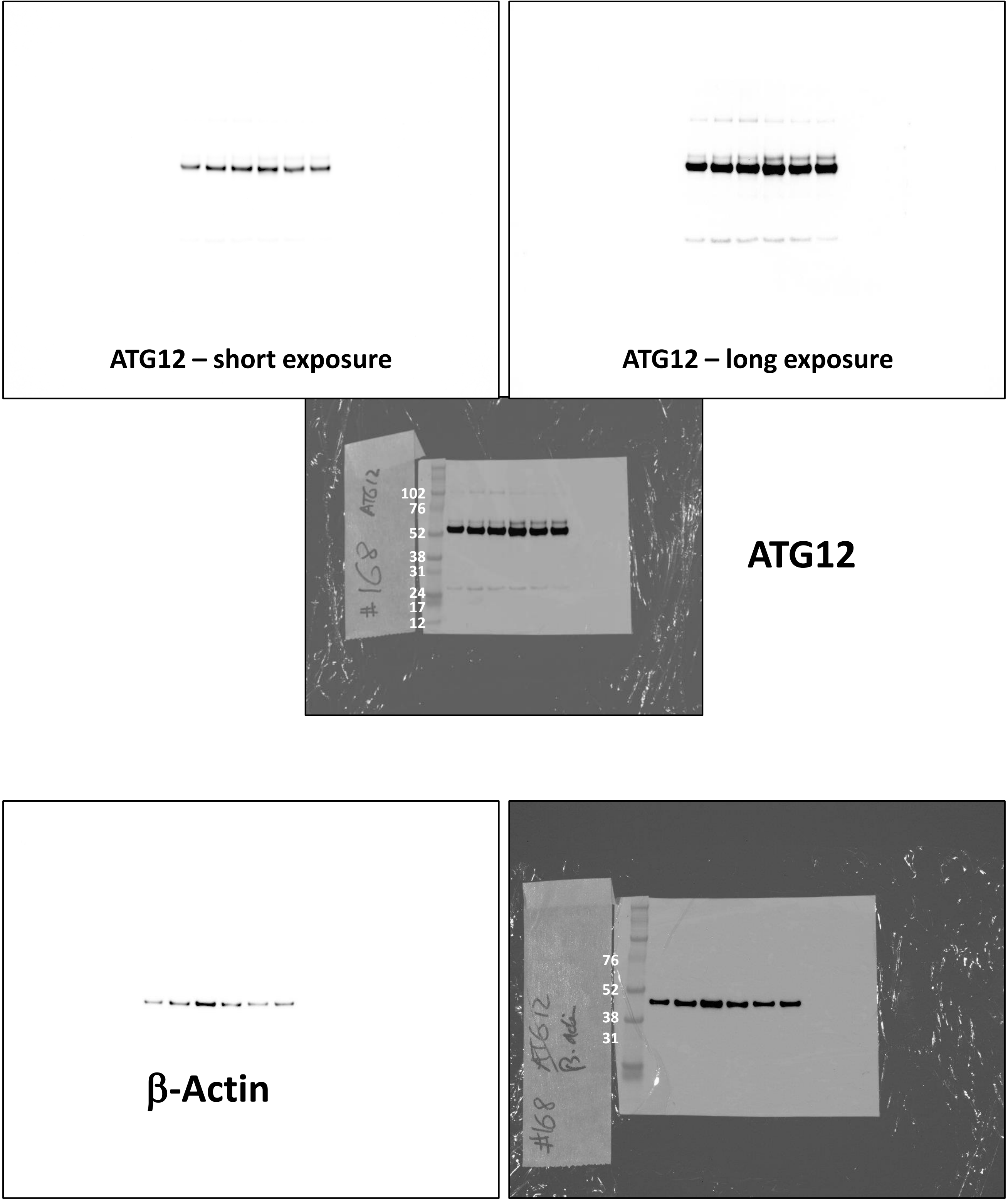

Supplementary Figure S12: Original blots for Figure 4c

This figure includes original blots, including multiple exposures for ATG12, as well as composite overlay images displaying molecular weight markers (relevant kDa values are indicated).

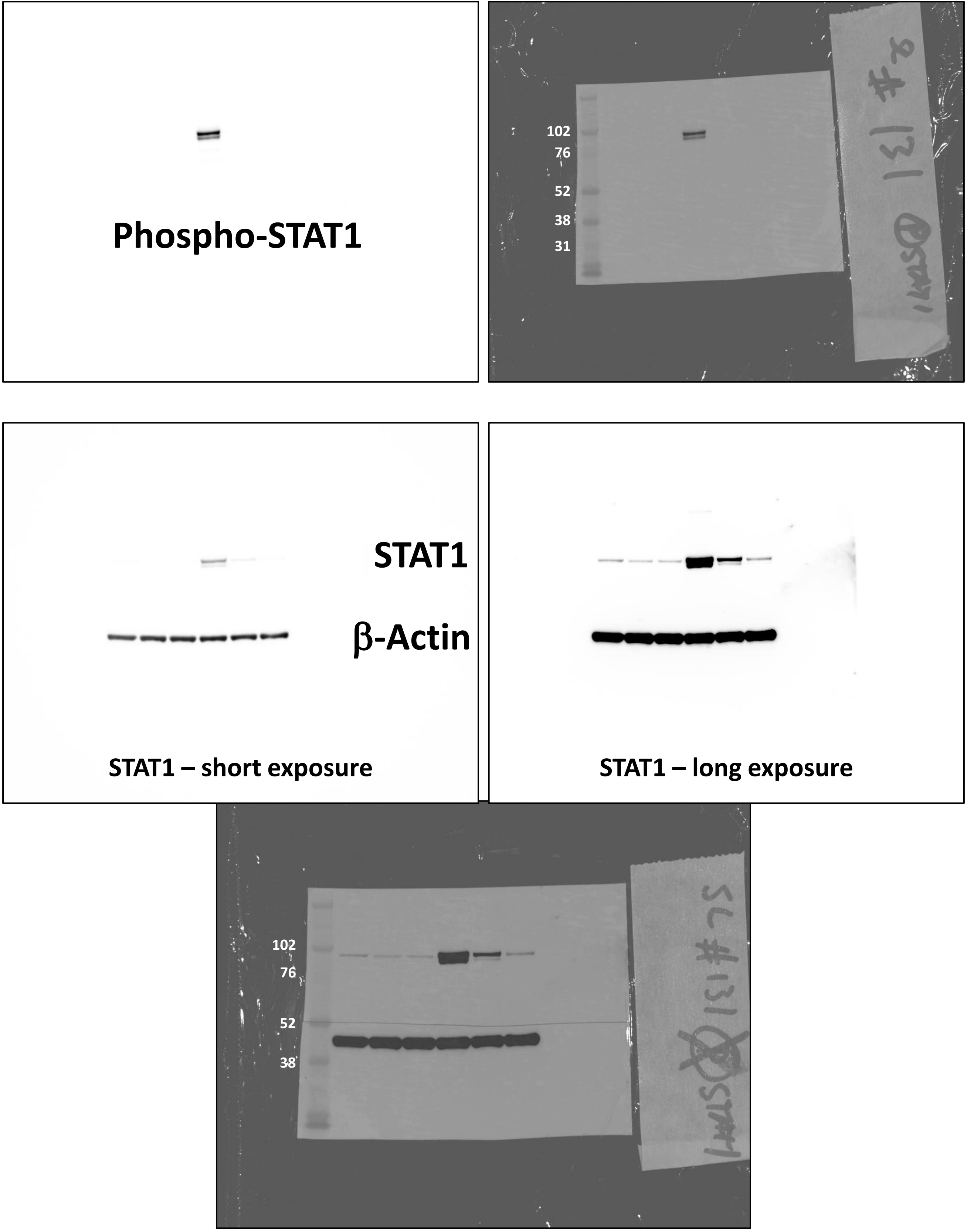

Supplementary Figure S13: Original blots for figure 5a
This figure includes original blots, including multiple exposures for STAT1/β-Actin blots, as well as composite overlay images displaying molecular weight markers (relevant kDa values are indicated).

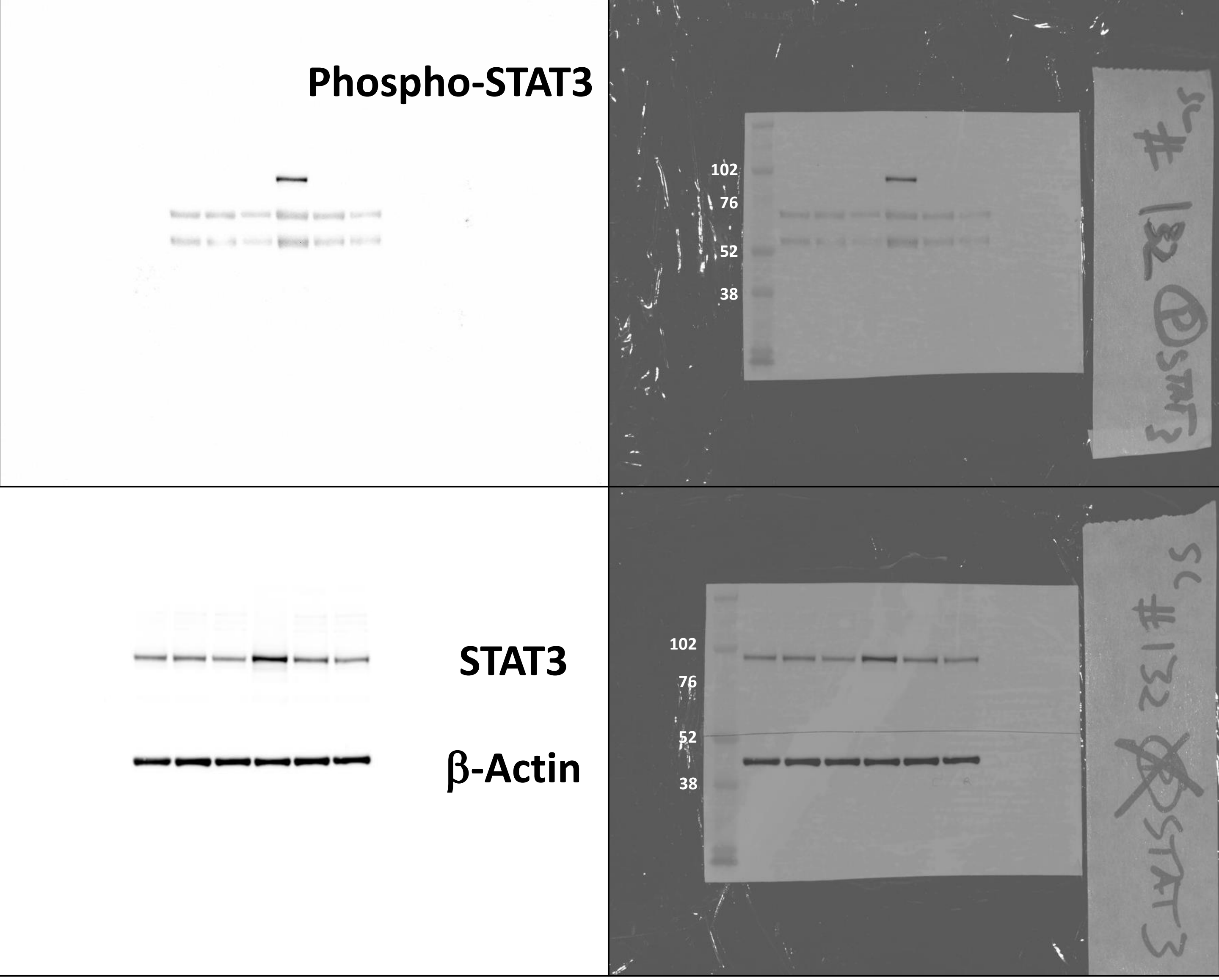

Supplementary Figure S14: Original blots for figure 5c
This figure includes original blots as well as composite overlay images displaying molecular weight markers (relevant kDa values are indicated).

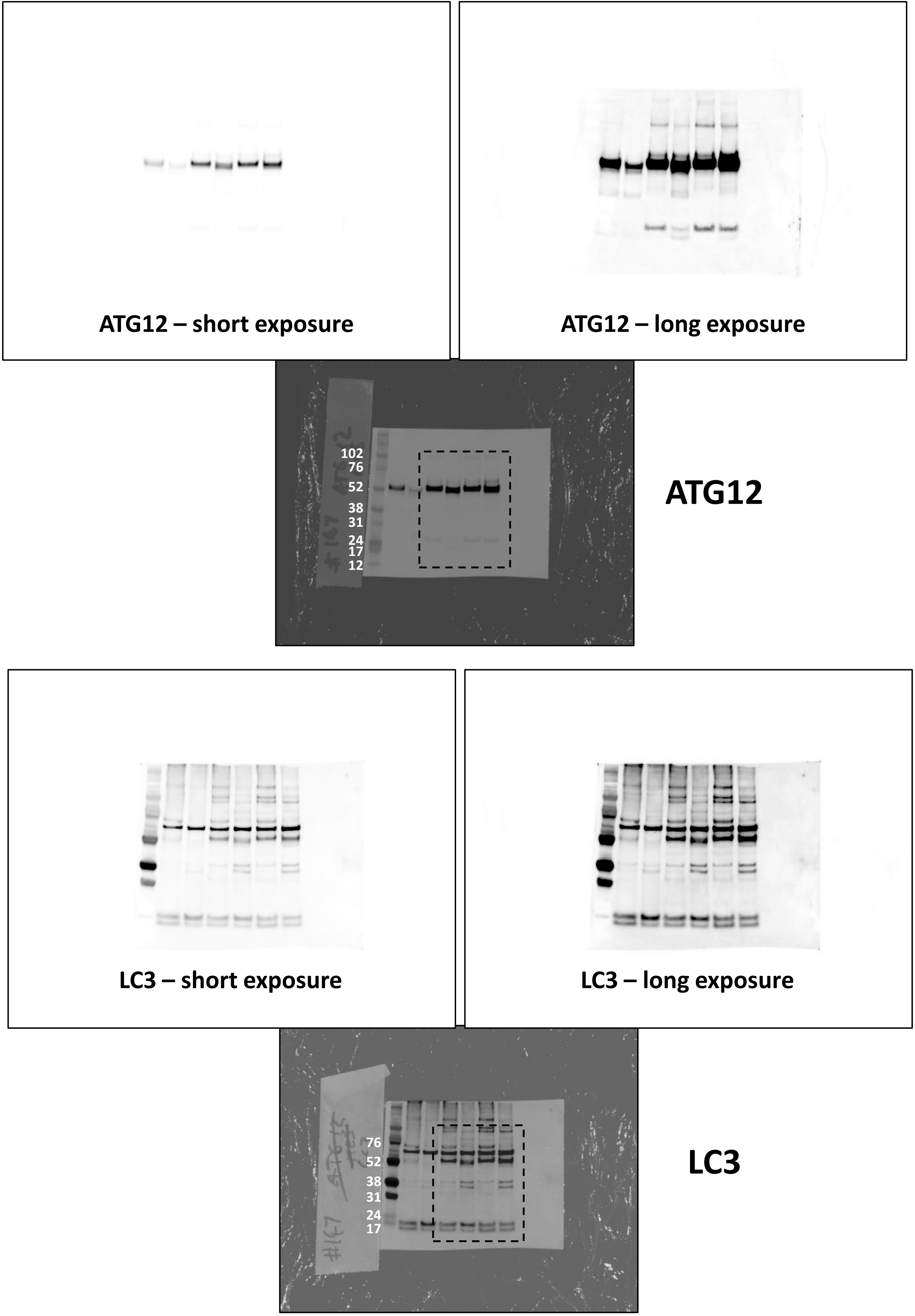

Supplementary Figure S15: Original blots for Supplementary figure S7
This figure includes original blots, including multiple exposures for ATG12 and LC3 blots, as well as composite overlay images displaying molecular weight markers (relevant kDa values are indicated). Only outlined lanes have been used in the final figure.

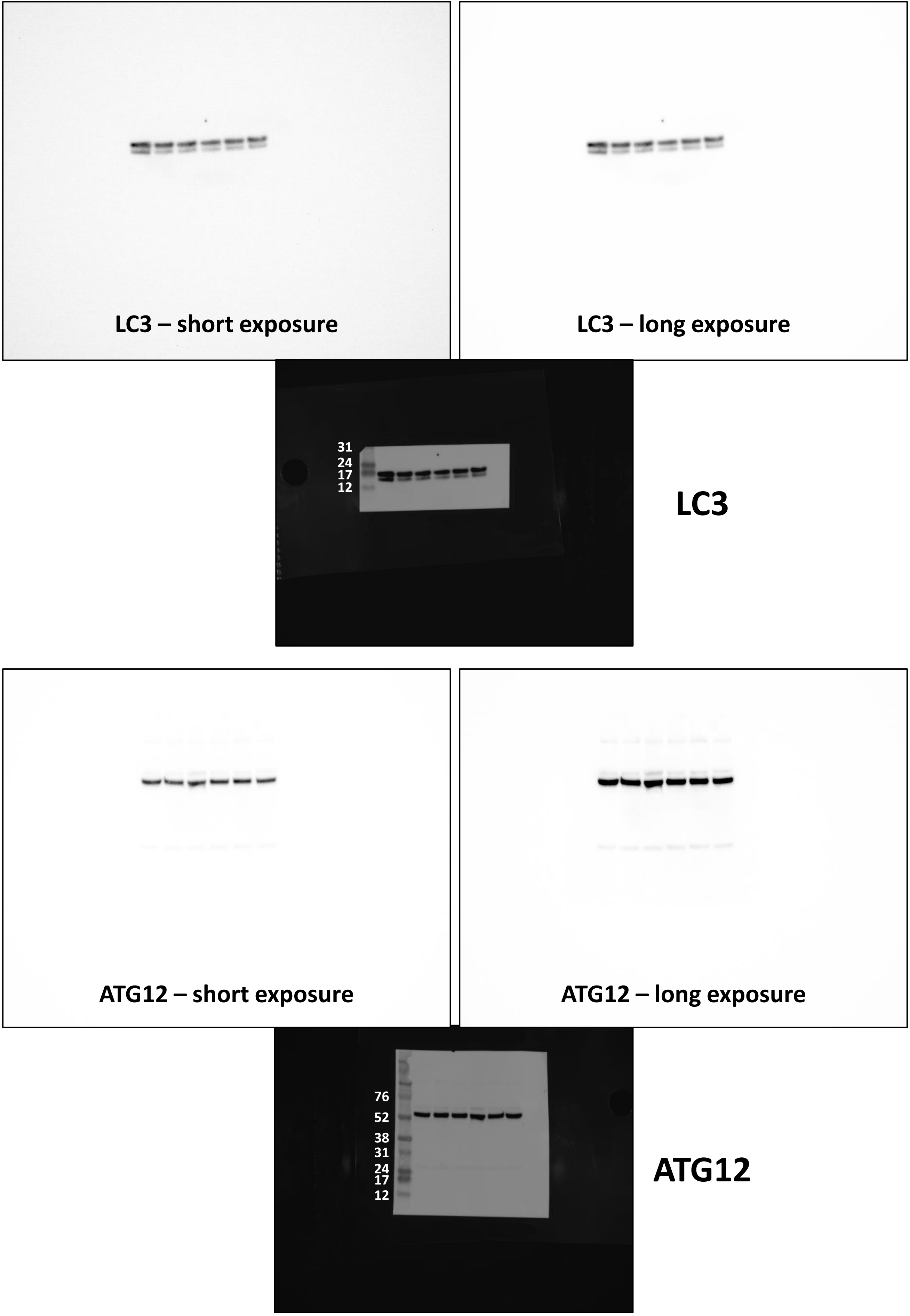

Supplementary Figure S16: Original blots for Supplementary figure S8
This figure includes original blots, including multiple exposures for ATG12 and LC3 blots, as well as composite overlay images displaying molecular weight markers (relevant kDa values are indicated).

Supplemental Table S1.

The expression profile of Autophagy-relatged genes (ATG) in ABI-MACs

ATG name (Gene Symbol)	F.C.*	SE	p-value
solute carrier family 7 member 11(SLC7A11)	3.54	1.56	0.079
NLR family pyrin domain containing 3(NLRP3)	1.54	1.13	0.011
napsin A aspartic peptidase(NAPSA)	1.50	1.12	0.030
desmin(DES)	1.48	1.36	0.319
nuclear factor, erythroid 2 like 3(NFE2L3)	1.44	1.35	0.369
runt related transcription factor 3(RUNX3)	1.43	1.09	0.032
ral guanine nucleotide dissociation stimulator like 1(RGL1)	1.40	1.12	0.082
sestrin 2(SESN2)	1.39	1.12	0.023
kinase suppressor of ras 1(KSR1)	1.35	1.09	0.078
Fos proto-oncogene, AP-1 transcription factor subunit(FOS)	1.35	1.09	0.080
ATP binding cassette subfamily B member 6 (Langereis blood	1.34	1.05	0.070
solute carrier family 1 member 5(SLC1A5)	1.34	1.18	0.112
heat shock protein family A (Hsp70) member 6(HSPA6)	1.34	1.09	0.012
mannosidase alpha class 2A member 2(MAN2A2)	1.33	1.08	0.011
pregnancy up-regulated nonubiquitous CaM kinase(PNCK)	1.31	1.11	0.023
autophagy related 16 like 2(ATG16L2)	1.30	1.15	0.113
adaptor related protein complex 4 epsilon 1 subunit(AP4E1)	1.30	1.12	0.357
phospholipase D2(PLD2)	1.30	1.18	0.139
autophagy related 2A(ATG2A)	1.29	1.16	0.073
insulin receptor(INSR)	1.29	1.08	0.043
kelch like family member 24(KLHL24)	1.28	1.08	0.040
calpain 3(CAPN3)	1.28	1.07	0.101
proliferation-associated 2G4(PA2G4)	1.27	1.15	0.293
solute carrier family 1 member 2(SLC1A2)	1.27	1.25	0.338
NLR family pyrin domain containing 12(NLRP12)	1.26	1.18	0.422
TNF alpha induced protein 2(TNFAIP2)	1.26	1.11	0.128
anaplastic lymphoma receptor tyrosine kinase(ALK)	1.26	1.07	0.049
NFKB inhibitor zeta(NFKBIZ)	1.26	1.16	0.111

heat shock protein family A (Hsp70) member 1B(HSPA1B)	1.25	1.02	0.041
dynamin 1(DNM1)	1.25	1.13	0.085
dynamin 1(DNM1)	1.25	1.13	0.085
PTEN induced putative kinase 1(PINK1)	1.25	1.07	0.018
NBR1, autophagy cargo receptor(NBR1)	1.24	1.06	0.041
solute carrier family 36 member 4(SLC36A4)	1.24	1.02	0.055
protein kinase N2(PKN2)	1.24	1.07	0.042
exocyst complex component 3(EXOC3)	1.24	1.09	0.030
SID1 transmembrane family member 1(SIDT1)	1.24	1.01	0.018
kelch like family member 28(KLHL28)	1.24	1.01	0.018
calcium/calmodulin dependent protein kinase I(CAMK1)	1.23	1.21	0.327
trafficking protein particle complex 5(TRAPPC5)	1.23	1.02	0.002
calpain 7(CAPN7)	1.22	1.05	0.007
TNF receptor associated factor 2(TRAF2)	1.22	1.14	0.178
receptor interacting serine/threonine kinase 4(RIPK4)	1.22	1.07	0.051
misshapen like kinase 1(MINK1)	1.22	1.09	0.116
TBC1 domain family member 5(TBC1D5)	1.22	1.04	0.006
cyclin dependent kinase 13(CDK13)	1.21	1.02	0.014
ankyrin repeat domain 44(ANKRD44)	1.21	1.10	0.103
alkaline phosphatase, intestinal(ALPI)	1.21	1.09	0.040
superoxide dismutase 2, mitochondrial(SOD2)	1.20	1.12	0.178
ubiquitin conjugating enzyme E2 N(UBE2N)	1.20	1.23	0.311
hypoxia inducible factor 3 alpha subunit(HIF3A)	1.20	1.05	0.019
heat shock protein family A (Hsp70) member 1A(HSPA1A)	1.20	1.03	0.053
ankyrin 1(ANK1)	1.20	1.09	0.072
calpain 8(CAPN8)	1.19	1.07	0.049
glyceraldehyde-3-phosphate dehydrogenase, spermatogenic(GAPDHS)	1.19	1.09	0.057
large tumor suppressor kinase 2(LATS2)	1.19	1.05	0.076
vacuole membrane protein 1(VMP1)	1.19	1.05	0.013
nucleotide binding oligomerization domain containing 1(NOD1)	1.19	1.08	0.041
actin, alpha 2, smooth muscle, aorta(ACTA2)	1.19	1.21	0.496
ATR serine/threonine kinase(ATR)	1.18	1.06	0.344
phosphatidylinositol-3,4,5-trisphosphate dependent Rac exchange factor 1(PREX1)	1.18	1.12	0.195
protein phosphatase 2 regulatory subunit B'beta(PPP2R5B)	1.18	1.10	0.231

NLR family pyrin domain containing 4(NLRP4)	1.18	1.03	0.069
cyclin dependent kinase inhibitor 1B(CDKN1B)	1.17	1.04	0.008
Protease-activated receptor-1 (par1)	1.17	1.01	0.007
salt inducible kinase 1(SIK1)	1.17	1.06	0.115
signal induced proliferation associated 1 like 3(SIPA1L3)	1.17	1.05	0.048
NFKB inhibitor alpha(NFKBIA)	1.17	1.11	0.350
insulin receptor substrate 2(IRS2)	1.17	1.08	0.110
protein kinase C zeta(PRKCZ)	1.17	1.09	0.062
SIN3 transcription regulator family member A(SIN3A)	1.17	1.05	0.048
nuclear factor, erythroid 2 like 1(NFE2L1)	1.17	1.09	0.167
myotubularin related protein 7(MTMR7)	1.17	1.06	0.242
forkhead box O3(FOXO3)	1.17	1.11	0.137
keratin 6A(KRT6A)	1.17	1.17	0.306
cathepsin E(CTSE)	1.17	1.12	0.212
p21 (RAC1) activated kinase 1(PAK1)	1.17	1.15	0.271
NLR family pyrin domain containing 1(NLRP1)	1.16	1.15	0.660
phosphatidylinositol-4-phosphate 5-kinase type 1 alpha(PIP5K1A)	1.16	1.05	0.072
nuclear receptor coactivator 4(NCOA4)	1.16	1.05	0.032
BLK proto-oncogene, Src family tyrosine kinase(BLK)	1.16	1.03	0.005
epsin 1(EPN1)	1.16	1.15	0.213
solute carrier family 7 member 7(SLC7A7)	1.16	1.04	0.072
exocyst complex component 7(EXOC7)	1.16	1.03	0.085
SPG7, paraplegin matrix AAA peptidase subunit(SPG7)	1.16	1.06	0.065
WD repeat domain, phosphoinositide interacting 2(WIPI2)	1.16	1.01	0.058
regulator of calcineurin 1(RCAN1)	1.16	1.09	0.374
heat shock protein family A (Hsp70) member 1 like(HSPA1L)	1.16	1.06	0.365
keratin 8(KRT8)	1.15	1.08	0.399
kelch like family member 12(KLHL12)	1.15	1.04	0.018
kelch like family member 8(KLHL8)	1.15	1.06	0.045
transmembrane 9 superfamily member 4(TM9SF4)	1.15	1.01	0.007
kelch like ECH associated protein 1(KEAP1)	1.15	1.06	0.196
NLR family pyrin domain containing 14(NLRP14)	1.15	1.12	0.161
eukaryotic translation initiation factor 4E binding protein 1(EIF4EBP1)	1.15	1.05	0.177
protein phosphatase 2 regulatory subunit Bdelta(PPP2R2D)	1.15	1.07	0.144

pepsinogen 3, group I (pepsinogen A)(PGA3)	1.15	1.18	0.321
lysine acetyltransferase 6A(KAT6A)	1.14	1.02	0.008
AKT1 substrate 1(AKT1S1)	1.14	1.05	0.192
MTOR associated protein, LST8 homolog(MLST8)	1.14	1.05	0.071
sirtuin 1(SIRT1)	1.14	1.02	0.002
insulin receptor substrate 1(IRS1)	1.14	1.15	0.405
runt related transcription factor 1(RUNX1)	1.14	1.07	0.076
RAB33B, member RAS oncogene family(RAB33B)	1.14	1.07	0.258
keratin 72(KRT72)	1.14	1.17	0.424
eukaryotic translation initiation factor 4E binding protein 2(EIF4EBP2)	1.13	1.05	0.030
UDP glucuronosyltransferase family 2 member A3(UGT2A3)	1.13	1.06	0.081
myotubularin related protein 9(MTMR9)	1.13	1.01	0.126
endoplasmic reticulum to nucleus signaling 1(ERN1)	1.13	1.01	0.005
protein kinase AMP-activated non-catalytic subunit beta 1(PRKAB1)	1.13	1.01	0.025
SIK family kinase 3(SIK3)	1.13	1.07	0.061
neuronal PAS domain protein 2(NPAS2)	1.13	1.06	0.089
casein kinase 2 alpha 2(CSNK2A2)	1.13	1.12	0.220
ATM serine/threonine kinase(ATM)	1.13	1.11	0.246
zinc finger FYVE-type containing 1(ZFYVE1)	1.12	1.03	0.066
solute carrier family 3 member 2(SLC3A2)	1.12	1.06	0.283
lysine acetyltransferase 7(KAT7)	1.12	1.05	0.044
VPS39, HOPS complex subunit(VPS39)	1.12	1.06	0.062
histone deacetylase 4(HDAC4)	1.12	1.08	0.219
WD repeat domain 5B(WDR5B)	1.12	1.06	0.368
RAB12, member RAS oncogene family(RAB12)	1.12	1.01	0.064
HCK proto-oncogene, Src family tyrosine kinase(HCK)	1.12	1.09	0.207
keratin 76(KRT76)	1.12	1.06	0.305
UDP glucuronosyltransferase family 1 member A9(UGT1A9)	1.12	1.06	0.101
SOS Ras/Rho guanine nucleotide exchange factor 2(SOS2)	1.12	1.06	0.111
protein kinase N3(PKN3)	1.12	1.09	0.267
mitogen-activated protein kinase kinase 2(MAP2K2)	1.12	1.09	0.467
SET binding factor 2(SBF2)	1.12	1.06	0.188
GRAM domain containing 1B(GRAMD1B)	1.12	1.06	0.591
calcium/calmodulin dependent protein kinase kinase 1(CAMKK1)	1.12	1.12	0.314

LCK proto-oncogene, Src family tyrosine kinase(LCK)	1.12	1.07	0.134
protein kinase N1(PKN1)	1.12	1.09	0.252
adaptor related protein complex 1 gamma 2 subunit(AP1G2)	1.12	1.11	0.403
alkaline phosphatase, placental(ALPP)	1.12	1.16	0.443
keratin 75(KRT75)	1.11	1.12	0.591
calpain small subunit 2(CAPNS2)	1.11	1.18	0.539
syntaxin binding protein 2(STXBP2)	1.11	1.04	0.022
RB1 inducible coiled-coil 1(RB1CC1)	1.11	1.12	0.292
ribosomal protein S6 kinase B2(RPS6KB2)	1.11	1.08	0.214
mitogen-activated protein kinase 3(MAPK3)	1.11	1.12	0.465
sequestosome 1(SQSTM1)	1.11	1.08	0.299
phosphatidylinositol 4-kinase beta(PI4KB)	1.11	1.06	0.162
lysine acetyltransferase 8(KAT8)	1.11	1.05	0.346
protein phosphatase 6 catalytic subunit(PPP6C)	1.11	1.05	0.195
UDP glucuronosyltransferase family 2 member B10(UGT2B10)	1.11	1.05	0.106
phosphatase and tensin homolog(PTEN)	1.11	1.13	0.370
kelch like family member 13(KLHL13)	1.11	1.06	0.128
nuclear factor, erythroid 2 like 2(NFE2L2)	1.11	1.02	0.142
calcium binding and coiled-coil domain 2(CALCOCO2)	1.11	1.00	0.064
WD repeat domain 45(WDR45)	1.11	1.05	0.080
death associated protein kinase 1(DAPK1)	1.10	1.09	0.247
mitogen-activated protein kinase kinase 5(MAP2K5)	1.10	1.08	0.169
synaptosome associated protein 29(SNAP29)	1.10	1.04	0.196
TNF receptor associated factor 3(TRAF3)	1.10	1.06	0.179
FUN14 domain containing 1(FUNDC1)	1.10	1.05	0.110
NUAK family kinase 2(NUAK2)	1.10	1.05	0.063
serine/threonine kinase 11(STK11)	1.10	1.06	0.163
adaptor related protein complex 2 alpha 2 subunit(AP2A2)	1.10	1.19	0.530
autophagy related 14(ATG14)	1.10	1.01	0.010
autophagy related 14(ATG14)	1.10	1.01	0.010
GABA type A receptor associated protein like 1(GABARAPL1)	1.10	1.04	0.450
adaptor related protein complex 1 sigma 2 subunit(AP1S2)	1.10	1.09	0.655
phosphatidylinositol-4-phosphate 5-kinase type 1 gamma(PIP5K1C)	1.10	1.06	0.181
autophagy related 16 like 1(ATG16L1)	1.10	1.06	0.303

protein kinase AMP-activated catalytic subunit alpha 2(PRKAA2)	1.10	1.06	0.424
aryl hydrocarbon receptor nuclear translocator(ARNT)	1.09	1.01	0.247
TBC1 domain family member 25(TBC1D25)	1.09	1.13	0.939
melanogenesis associated transcription factor(MITF)	1.09	1.01	0.070
Rho GTPase activating protein 26(ARHGAP26)	1.09	1.14	0.579
SGK2, serine/threonine kinase 2(SGK2)	1.09	1.15	0.490
neuronal PAS domain protein 3(NPAS3)	1.09	1.10	0.443
Rho guanine nucleotide exchange factor 4(ARHGEF4)	1.09	1.05	0.232
A-Raf proto-oncogene, serine/threonine kinase(ARAF)	1.09	1.02	0.287
adaptor related protein complex 4 mu 1 subunit(AP4M1)	1.09	1.07	0.274
SH3 domain containing GRB2 like 3, endophilin A3(SH3GL3)	1.09	1.07	0.320
telomerase associated protein 1(TEP1)	1.08	1.14	0.470
ABL proto-oncogene 1, non-receptor tyrosine kinase(ABL1)	1.08	1.03	0.024
DNA damage inducible transcript 4(DDIT4)	1.08	1.30	0.768
exocyst complex component 3 like 1(EXOC3L1)	1.08	1.00	0.020
SNF related kinase(SNRK)	1.08	1.04	0.310
autophagy related 9A(ATG9A)	1.08	1.03	0.330
erb-b2 receptor tyrosine kinase 3(ERBB3)	1.08	1.04	0.041
amino-terminal enhancer of split(AES)	1.08	1.08	0.585
phospholipase C beta 4(PLCB4)	1.08	1.06	0.189
dynamin 1 like(DNM1L)	1.08	1.06	0.239
phosphoinositide-3-kinase regulatory subunit 4(PIK3R4)	1.08	1.04	0.283
WW domain containing adaptor with coiled-coil(WAC)	1.08	1.04	0.127
protein arginine methyltransferase 8(PRMT8)	1.08	1.13	0.546
NFKB inhibitor beta(NFKBIB)	1.08	1.05	0.588
clathrin light chain B(CLTB)	1.08	1.03	0.581
phospholipase C eta 1(PLCH1)	1.07	1.09	0.469
trafficking protein particle complex 10(TRAPPC10)	1.07	1.06	0.240
GABA type A receptor associated protein like 3 pseudogene(GABARAPL3)	1.07	1.07	0.402
UDP glucuronosyltransferase family 2 member B28(UGT2B28)	1.07	1.09	0.556
calpain 12(CAPN12)	1.07	1.03	0.126
sorting nexin family member 30(SNX30)	1.07	1.01	0.185
protein phosphatase 2 regulatory subunit B'gamma(PPP2R5C)	1.07	1.07	0.518
endoplasmic reticulum to nucleus signaling 2(ERN2)	1.07	1.02	0.230

RELB proto-oncogene, NF-kB subunit(RELB)	1.07	1.05	0.159
TBC1 domain family member 14(TBC1D14)	1.07	1.07	0.360
TANK binding kinase 1(TBK1)	1.07	1.03	0.292
mitogen-activated protein kinase kinase 7(MAP2K7)	1.07	1.07	0.521
hormonally up-regulated Neu-associated kinase(HUNK)	1.07	1.02	0.105
protein kinase C theta(PRKCQ)	1.07	1.06	0.291
UV radiation resistance associated(UVRAG)	1.07	1.05	0.448
inositol 1,4,5-trisphosphate receptor type 3(ITPR3)	1.07	1.06	0.629
Raf-1 proto-oncogene, serine/threonine kinase(RAF1)	1.07	1.03	0.112
NLR family pyrin domain containing 13(NLRP13)	1.07	1.04	0.292
beclin 1(BECN1)	1.07	1.03	0.078
autophagy related 2B(ATG2B)	1.07	1.05	0.399
vacuolar protein sorting 45 homolog(VPS45)	1.06	1.07	0.600
keratin 71(KRT71)	1.06	1.13	0.584
calcium/calmodulin dependent protein kinase ID(CAMK1D)	1.06	1.17	0.692
serine/threonine kinase 38 like(STK38L)	1.06	1.15	0.656
mitogen-activated protein kinase kinase kinase kinase 1(MAP4K1)	1.06	1.15	0.595
C-type lectin domain family 16 member A(CLEC16A)	1.06	1.02	0.560
transmembrane phosphoinositide 3-phosphatase and tensin homolog 2(TPTE2)	1.06	1.09	0.518
ryanodine receptor 1(RYR1)	1.06	1.06	0.313
NLR family CARD domain containing 5(NLRC5)	1.06	1.08	0.613
phospholipase C eta 2(PLCH2)	1.06	1.03	0.132
fibroblast growth factor receptor 2(FGFR2)	1.06	1.05	0.410
mitogen-activated protein kinase kinase 4(MAP2K4)	1.06	1.11	0.543
solute carrier family 36 member 1(SLC36A1)	1.06	1.15	0.647
mechanistic target of rapamycin(MTOR)	1.06	1.04	0.354
ubiquitin C(UBC)	1.06	1.06	0.683
SEC16 homolog B, endoplasmic reticulum export factor(SEC16B)	1.06	1.12	0.609
unc-51 like autophagy activating kinase 2(ULK2)	1.06	1.10	0.561
unc-51 like autophagy activating kinase 1(ULK1)	1.05	1.05	0.301
protein phosphatase 2 regulatory subunit B''beta(PPP2R3B)	1.05	1.05	0.230
death associated protein kinase 3(DAPK3)	1.05	1.05	0.352
UDP glucuronosyltransferase family 2 member B15(UGT2B15)	1.05	1.05	0.313
myotubularin 1(MTM1)	1.05	1.04	0.454

torsin family 1 member B(TOR1B)	1.05	1.05	0.240
adaptor related protein complex 1 mu 2 subunit(AP1M2)	1.05	1.12	0.571
inositol 1,4,5-trisphosphate receptor type 2(ITPR2)	1.05	1.05	0.439
microtubule associated protein 1 light chain 3 alpha(MAP1LC3A)	1.05	1.08	0.528
mitochondrial translational release factor 1 like(MTRF1L)	1.05	1.13	0.585
ArfGAP with coiled-coil, ankyrin repeat and PH domains 3(ACAP3)	1.05	1.06	0.458
cathepsin B(CTSB)	1.05	1.03	0.172
calpain 1(CAPN1)	1.05	1.02	0.313
microtubule affinity regulating kinase 2(MARK2)	1.05	1.06	0.606
translocated promoter region, nuclear basket protein(TPR)	1.05	1.02	0.262
adaptor related protein complex 3 mu 2 subunit(AP3M2)	1.05	1.22	0.713
CUGBP, Elav-like family member 3(CELF3)	1.05	1.14	0.625
phosphatidylinositol-4,5-bisphosphate 3-kinase catalytic subunit alpha(PIK3CA)	1.05	1.07	0.522
SEC16 homolog A, endoplasmic reticulum export factor(SEC16A)	1.05	1.01	0.250
calpain 6(CAPN6)	1.05	1.06	0.606
valosin containing protein(VCP)	1.05	1.04	0.279
BR serine/threonine kinase 2(BRSK2)	1.05	1.10	0.519
protein phosphatase 2 catalytic subunit beta(PPP2CB)	1.05	1.04	0.455
SH3 domain containing GRB2 like 2, endophilin A1(SH3GL2)	1.05	1.03	0.579
secretion associated Ras related GTPase 1B(SAR1B)	1.05	1.10	0.587
eukaryotic translation initiation factor 4E family member 1B(EIF4E1B)	1.05	1.08	0.553
EPH receptor A2(EPHA2)	1.05	1.09	0.555
Ras related GTP binding B(RRAGB)	1.05	1.06	0.639
syntaxin binding protein 3(STXBP3)	1.05	1.03	0.320
autophagy related 4D cysteine peptidase(ATG4D)	1.05	1.09	0.592
adaptor related protein complex 1 gamma 1 subunit(AP1G1)	1.05	1.02	0.388
tuberous sclerosis 1(TSC1)	1.05	1.03	0.495
ubiquitin conjugating enzyme E2 D2(UBE2D2)	1.05	1.13	0.720
VPS33B, late endosome and lysosome associated(VPS33B)	1.04	1.04	0.595
eukaryotic translation elongation factor 1 alpha 2(EEF1A2)	1.04	1.15	0.679
VPS11, CORVET/HOPS core subunit(VPS11)	1.04	1.05	0.398
nucleotide binding oligomerization domain containing 2(NOD2)	1.04	1.21	0.751
epsin 3(EPN3)	1.04	1.08	0.533
transformation/transcription domain associated protein(TRRAP)	1.04	1.06	0.405

adaptor related protein complex 4 beta 1 subunit(AP4B1)	1.04	1.04	0.479
unc-51 like kinase 3(ULK3)	1.04	1.08	0.577
RAS like proto-oncogene A(RALA)	1.04	1.22	0.822
MER proto-oncogene, tyrosine kinase(MERTK)	1.04	1.05	0.542
adaptor related protein complex 1 mu 1 subunit(AP1M1)	1.04	1.09	0.652
ribosomal protein S6 kinase A6(RPS6KA6)	1.04	1.01	0.205
calpain 13(CAPN13)	1.04	1.06	0.548
NFKB inhibitor epsilon(NFKBIE)	1.04	1.09	0.758
autophagy related 4C cysteine peptidase(ATG4C)	1.04	1.05	0.486
keratin 85(KRT85)	1.04	1.07	0.531
WD repeat domain 5(WDR5)	1.04	1.11	0.696
ribosomal protein S6 kinase B1(RPS6KB1)	1.04	1.05	0.442
fission, mitochondrial 1(FIS1)	1.04	1.02	0.487
tuberous sclerosis 2(TSC2)	1.04	1.03	0.150
tuberous sclerosis 2(TSC2)	1.04	1.03	0.150
serine/threonine kinase 4(STK4)	1.04	1.03	0.143
histone deacetylase 6(HDAC6)	1.04	1.14	0.721
Rho guanine nucleotide exchange factor 17(ARHGEF17)	1.04	1.19	0.866
VPS33A, CORVET/HOPS core subunit(VPS33A)	1.04	1.06	0.549
protein phosphatase 2 regulatory subunit B'epsilon(PPP2R5E)	1.04	1.06	0.465
autophagy related 7(ATG7)	1.03	1.09	0.673
autophagy related 7(ATG7)	1.03	1.09	0.673
autophagy related 7(ATG7)	1.03	1.09	0.673
autophagy related 7(ATG7)	1.03	1.09	0.673
MX dynamin like GTPase 1(MX1)	1.03	1.23	0.952
SRC proto-oncogene, non-receptor tyrosine kinase(SRC)	1.03	1.09	0.692
protein kinase C beta(PRKCB)	1.03	1.02	0.774
aryl hydrocarbon receptor nuclear translocator like 2(ARNTL2)	1.03	1.03	0.737
p21 (RAC1) activated kinase 2(PAK2)	1.03	1.06	0.724
insulin receptor related receptor(INSRR)	1.03	1.01	0.194
karyopherin subunit alpha 6(KPNA6)	1.03	1.02	0.661
myosin IIIA(MYO3A)	1.03	1.03	0.611
actin, gamma 2, smooth muscle, enteric(ACTG2)	1.03	1.06	0.747
ribosomal protein S6 kinase A4(RPS6KA4)	1.03	1.19	0.873

SEC24 homolog D, COPII coat complex component(SEC24D)	1.03	1.02	0.295
AKT serine/threonine kinase 1(AKT1)	1.03	1.05	0.594
EPH receptor B3(EPHB3)	1.03	1.09	0.709
trafficking protein particle complex 6A(TRAPPC6A)	1.03	1.07	0.635
serine/threonine kinase 25(STK25)	1.03	1.06	0.666
BCR, RhoGEF and GTPase activating protein(BCR)	1.03	1.08	0.630
NLR family CARD domain containing 3(NLRC3)	1.03	1.08	0.884
TBC1 domain family member 7(TBC1D7)	1.03	1.03	0.709
serine/threonine kinase 36(STK36)	1.03	1.10	0.826
SEC24 homolog C, COPII coat complex component(SEC24C)	1.03	1.02	0.756
KRAS proto-oncogene, GTPase(KRAS)	1.03	1.06	0.709
myotubularin related protein 6(MTMR6)	1.03	1.02	0.777
protein phosphatase 2 catalytic subunit alpha(PPP2CA)	1.03	1.07	0.732
SH3 domain containing GRB2 like endophilin B2(SH3GLB2)	1.03	1.04	0.621
cyclin dependent kinase 11A(CDK11A)	1.03	1.10	0.706
RPTOR independent companion of MTOR complex 2(RICTOR)	1.03	1.06	0.678
aspartyl aminopeptidase(DNPEP)	1.02	1.07	0.652
trafficking protein particle complex 6B(TRAPPC6B)	1.02	1.08	0.717
AKT serine/threonine kinase 3(AKT3)	1.02	1.17	0.927
proline rich 5 like(PRR5L)	1.02	1.32	0.810
NLR family pyrin domain containing 8(NLRP8)	1.02	1.05	0.549
growth factor receptor bound protein 2(GRB2)	1.02	1.01	0.227
superoxide dismutase 1, soluble(SOD1)	1.02	1.08	0.725
neurotrophic receptor tyrosine kinase 2(NTRK2)	1.02	1.06	0.693
actin, alpha, cardiac muscle 1(ACTC1)	1.02	1.08	0.730
adaptor related protein complex 2 alpha 1 subunit(AP2A1)	1.02	1.11	0.829
ADP ribosylation factor guanine nucleotide exchange factor 1(ARFGEF1)	1.02	1.05	0.678
ArfGAP with coiled-coil, ankyrin repeat and PH domains 2(ACAP2)	1.02	1.06	0.751
trafficking protein particle complex 9(TRAPPC9)	1.02	1.05	0.790
trafficking protein particle complex 4(TRAPPC4)	1.02	1.11	0.822
cyclin dependent kinase 17(CDK17)	1.02	1.03	0.853
histone deacetylase 5(HDAC5)	1.02	1.05	0.778
RELA proto-oncogene, NF-kB subunit(RELA)	1.02	1.02	0.813
autophagy related 3(ATG3)	1.02	1.02	0.739

autophagy related 3(ATG3)	1.02	1.02	0.739
myotubularin related protein 8(MTMR8)	1.02	1.25	0.960
forkhead box O1(FOXO1)	1.02	1.00	0.570
ankyrin 3(ANK3)	1.02	1.03	0.730
keratin 84(KRT84)	1.02	1.01	0.465
EPH receptor A5(EPHA5)	1.01	1.03	0.695
E2F transcription factor 2(E2F2)	1.01	1.10	0.947
cyclin dependent kinase like 1(CDKL1)	1.01	1.08	0.790
keratin 81(KRT81)	1.01	1.14	0.932
eukaryotic translation elongation factor 1 alpha 1(EEF1A1)	1.01	1.06	0.781
vesicle associated membrane protein 8(VAMP8)	1.01	1.06	0.879
SEC24 homolog B, COPII coat complex component(SEC24B)	1.01	1.00	0.723
nuclear receptor binding factor 2(NRBF2)	1.01	1.01	0.737
vesicle transport through interaction with t-SNAREs 1B(VTI1B)	1.01	1.04	0.873
adaptor related protein complex 1 beta 1 subunit(AP1B1)	1.01	1.06	0.834
pepsinogen 4, group I (pepsinogen A)(PGA4)	1.01	1.06	0.850
NLR family pyrin domain containing 7(NLRP7)	1.01	1.09	0.932
casein kinase 1 gamma 2(CSNK1G2)	1.01	1.03	0.783
Sec23 homolog B, coat complex II component(SEC23B)	1.01	1.04	0.848
mitogen-activated protein kinase 1(MAPK1)	1.01	1.01	0.765
mitogen-activated protein kinase 8 interacting protein 1(MAPK8IP1)	1.01	1.11	0.875
mannosidase alpha class 2C member 1(MAN2C1)	1.01	1.12	0.969
DEP domain containing MTOR-interacting protein(DEPTOR)	1.01	1.10	0.943
ADP ribosylation factor guanine nucleotide exchange factor 2(ARFGEF2)	1.01	1.10	0.881
syntaxin binding protein 1(STXBP1)	1.01	1.22	0.932
exocyst complex component 8(EXOC8)	1.01	1.03	0.925
EI24, autophagy associated transmembrane protein(EI24)	1.00	1.05	0.979
exocyst complex component 1(EXOC1)	1.00	1.02	0.912
EPH receptor A3(EPHA3)	1.00	1.05	0.927
caspase 3(CASP3)	1.00	1.03	0.969
kelch like family member 2(KLHL2)	1.00	1.17	0.904
AKT serine/threonine kinase 2(AKT2)	1.00	1.02	0.944
oligophrenin 1(OPHN1)	1.00	1.08	0.960
TBC1 domain family member 12(TBC1D12)	1.00	1.18	1.000

lysine acetyltransferase 6B(KAT6B)	1.00	1.01	0.988
protein phosphatase 1 catalytic subunit gamma(PPP1CC)	-1.00	1.02	0.965
histone deacetylase 1(HDAC1)	-1.00	1.06	0.988
protein phosphatase 2 regulatory subunit Bbeta(PPP2R2B)	-1.00	1.02	0.991
mitogen-activated protein kinase 7(MAPK7)	-1.00	1.08	0.960
calpain 2(CAPN2)	-1.00	1.15	0.986
adaptor related protein complex 2 beta 1 subunit(AP2B1)	-1.00	1.02	0.925
NLR family pyrin domain containing 9(NLRP9)	-1.00	1.07	0.980
mitofusin 2(MFN2)	-1.00	1.13	0.948
solute carrier family 3 member 1(SLC3A1)	-1.00	1.05	0.918
erb-b2 receptor tyrosine kinase 2(ERBB2)	-1.00	1.05	0.912
cyclin dependent kinase inhibitor 2A(CDKN2A)	-1.01	1.03	0.978
fibroblast growth factor receptor 3(FGFR3)	-1.01	1.08	0.885
immunity related GTPase M(IRGM)	-1.01	1.09	0.932
nuclear factor kappa B subunit 1(NFKB1)	-1.01	1.04	0.934
peroxisomal biogenesis factor 1(PEX1)	-1.01	1.04	0.863
calcium/calmodulin dependent protein kinase IG(CAMK1G)	-1.01	1.04	0.872
kelch like family member 4(KLHL4)	-1.01	1.03	0.735
single-minded family bHLH transcription factor 2(SIM2)	-1.01	1.02	0.703
ubiquitin conjugating enzyme E2 D3(UBE2D3)	-1.01	1.03	0.837
casein kinase 2 beta(CSNK2B)	-1.01	1.02	0.904
AXL receptor tyrosine kinase(AXL)	-1.01	1.04	0.921
DIRAS family GTPase 3(DIRAS3)	-1.01	1.02	0.630
cyclin dependent kinase like 4(CDKL4)	-1.01	1.12	0.965
lysine acetyltransferase 5(KAT5)	-1.01	1.06	0.859
transmembrane 9 superfamily member 3(TM9SF3)	-1.01	1.10	0.940
histone deacetylase 7(HDAC7)	-1.01	1.07	0.839
p21 (RAC1) activated kinase 3(PAK3)	-1.01	1.05	0.834
GABA type A receptor-associated protein(GABARAP)	-1.01	1.03	0.740
exocyst complex component 4(EXOC4)	-1.01	1.07	0.994
casein kinase 1 delta(CSNK1D)	-1.01	1.07	0.829
receptor interacting serine/threonine kinase 1(RIPK1)	-1.01	1.01	0.750
harbinger transposase derived 1(HARBI1)	-1.01	1.08	0.970
calcium/calmodulin dependent protein kinase II gamma(CAMK2G)	-1.01	1.12	0.930

microtubule affinity regulating kinase 3(MARK3)	-1.01	1.01	0.868
activating transcription factor 4(ATF4)	-1.02	1.09	0.802
protein arginine methyltransferase 1(PRMT1)	-1.02	1.04	0.820
microtubule affinity regulating kinase 4(MARK4)	-1.02	1.03	0.724
Ras homolog enriched in brain(RHEB)	-1.02	1.14	0.935
TNF receptor associated factor 6(TRAF6)	-1.02	1.10	0.904
trafficking protein particle complex 3(TRAPPC3)	-1.02	1.05	0.715
autophagy related 5(ATG5)	-1.02	1.04	0.713
autophagy related 5(ATG5)	-1.02	1.04	0.713
autophagy related 5(ATG5)	-1.02	1.04	0.713
golgi brefeldin A resistant guanine nucleotide exchange factor 1(GBF1)	-1.02	1.06	0.708
protein kinase C iota(PRKCI)	-1.02	1.09	0.967
Ral GTPase activating protein catalytic alpha subunit 1(RALGAPA1)	-1.02	1.02	0.564
vacuolar protein sorting 13 homolog C(VPS13C)	-1.02	1.08	0.814
Sec23 homolog A, coat complex II component(SEC23A)	-1.02	1.01	0.096
EPH receptor A4(EPHA4)	-1.02	1.05	0.897
calpain 9(CAPN9)	-1.02	1.07	0.755
RAB5A, member RAS oncogene family(RAB5A)	-1.02	1.08	0.727
trafficking protein particle complex 8(TRAPPC8)	-1.02	1.04	0.621
microtubule affinity regulating kinase 1(MARK1)	-1.02	1.06	0.716
protein phosphatase 2 regulatory subunit Bgamma(PPP2R2C)	-1.02	1.08	0.765
RAB5C, member RAS oncogene family(RAB5C)	-1.02	1.05	0.706
regulator of G-protein signaling 19(RGS19)	-1.02	1.09	0.721
leucine rich repeat and sterile alpha motif containing 1(LRSAM1)	-1.02	1.10	0.829
P21 (RAC1) Activated Kinase 7 (PAK7)	-1.03	1.07	0.792
ryanodine receptor 2(RYR2)	-1.03	1.04	0.433
ankyrin repeat and kinase domain containing 1(ANKK1)	-1.03	1.17	0.836
conserved helix-loop-helix ubiquitous kinase(CHUK)	-1.03	1.09	0.706
protein phosphatase 2 regulatory subunit Balpha(PPP2R2A)	-1.03	1.12	0.852
UDP glucuronosyltransferase family 2 member B4(UGT2B4)	-1.03	1.08	0.659
SHC adaptor protein 4(SHC4)	-1.03	1.02	0.462
SOS Ras/Rac guanine nucleotide exchange factor 1(SOS1)	-1.03	1.02	0.235
myotubularin related protein 14(MTMR14)	-1.03	1.04	0.562
CUGBP, Elav-like family member 5(CELF5)	-1.03	1.02	0.834

small nuclear ribonucleoprotein U5 subunit 40(SNRNP40)	-1.03	1.06	0.692
pepsinogen 5, group I (pepsinogen A)(PGA5)	-1.03	1.14	0.787
phosphatidylinositol-5-phosphate 4-kinase type 2 alpha(PIP4K2A)	-1.03	1.03	0.655
RAB9A, member RAS oncogene family(RAB9A)	-1.03	1.13	0.994
exocyst complex component 2(EXOC2)	-1.03	1.05	0.701
protein phosphatase 2 regulatory subunit B''gamma(PPP2R3C)	-1.03	1.09	0.690
Ras related GTP binding A(RRAGA)	-1.03	1.14	0.911
sphingomyelin phosphodiesterase acid like 3B(SMPDL3B)	-1.03	1.05	0.615
ribosomal protein S6 kinase A1(RPS6KA1)	-1.03	1.02	0.444
FK506 binding protein 8(FKBP8)	-1.03	1.11	0.824
phosphatidylinositol 3-kinase catalytic subunit type 3(PIK3C3)	-1.03	1.11	0.802
BR serine/threonine kinase 1(BRSK1)	-1.03	1.14	0.719
AFG3 like matrix AAA peptidase subunit 2(AFG3L2)	-1.04	1.04	0.780
SIN3 transcription regulator family member B(SIN3B)	-1.04	1.09	0.612
transmembrane 9 superfamily member 1(TM9SF1)	-1.04	1.03	0.473
DNA damage regulated autophagy modulator 2(DRAM2)	-1.04	1.07	0.679
translocase of outer mitochondrial membrane 7(TOMM7)	-1.04	1.10	0.618
Rho GTPase activating protein 42(ARHGAP42)	-1.04	1.03	0.408
transcription factor binding to IGHM enhancer 3(TFE3)	-1.04	1.05	0.368
ARP3 actin related protein 3 homolog B(ACTR3B)	-1.04	1.31	0.878
transmembrane 9 superfamily member 2(TM9SF2)	-1.04	1.01	0.007
TATA-box binding protein associated factor 1(TAF1)	-1.04	1.03	0.854
solute carrier family 7 member 10(SLC7A10)	-1.04	1.04	0.709
keratin 82(KRT82)	-1.04	1.02	0.313
ArfGAP with FG repeats 1(AGFG1)	-1.04	1.08	0.536
RAB8A, member RAS oncogene family(RAB8A)	-1.04	1.05	0.356
p21 (RAC1) activated kinase 4(PAK4)	-1.04	1.11	0.612
autophagy related 4B cysteine peptidase(ATG4B)	-1.04	1.04	0.698
YES proto-oncogene 1, Src family tyrosine kinase(YES1)	-1.04	1.06	0.539
kelch like family member 18(KLHL18)	-1.04	1.06	0.606
acyl-CoA binding domain containing 5(ACBD5)	-1.04	1.07	0.684
heat shock protein family A (Hsp70) member 8(HSPA8)	-1.04	1.06	0.574
glyceraldehyde-3-phosphate dehydrogenase(GAPDH)	-1.04	1.07	0.419
UDP glucuronosyltransferase family 2 member B17(UGT2B17)	-1.04	1.12	0.651

PGAM family member 5, mitochondrial serine/threonine protein phosphatase(PGAM5)	-1.04	1.03	0.751
voltage dependent anion channel 3(VDAC3)	-1.04	1.08	0.615
B-Raf proto-oncogene, serine/threonine kinase(BRAF)	-1.05	1.04	0.207
dynamin 2(DNM2)	-1.05	1.06	0.450
lipin 2(LPIN2)	-1.05	1.05	0.245
adaptor related protein complex 1 associated regulatory protein(AP1AR)	-1.05	1.09	0.740
F-box and WD repeat domain containing 7(FBXW7)	-1.05	1.08	0.556
mitogen-activated protein kinase 14(MAPK14)	-1.05	1.06	0.476
lysosomal associated membrane protein 2(LAMP2)	-1.05	1.03	0.628
regulatory associated protein of MTOR complex 1(RPTOR)	-1.05	1.04	0.226
telomere maintenance 2(TELO2)	-1.05	1.07	0.613
solute carrier family 7 member 9(SLC7A9)	-1.05	1.05	0.373
tectonin beta-propeller repeat containing 1(TECPR1)	-1.05	1.02	0.367
brain derived neurotrophic factor(BDNF)	-1.05	1.03	0.406
EPH receptor B2(EPHB2)	-1.05	1.11	0.758
peroxisomal biogenesis factor 5 like(PEX5L)	-1.05	1.09	0.627
calpain 10(CAPN10)	-1.05	1.21	0.655
NFKB inhibitor delta(NFKBID)	-1.05	1.11	0.850
ARP2 actin related protein 2 homolog(ACTR2)	-1.06	1.03	0.159
OPA1, mitochondrial dynamin like GTPase(OPA1)	-1.06	1.06	0.599
kelch like family member 5(KLHL5)	-1.06	1.10	0.537
Ral GTPase activating protein catalytic alpha subunit 2(RALGAPA2)	-1.06	1.02	0.407
eukaryotic translation initiation factor 2 alpha kinase 1(EIF2AK1)	-1.06	1.06	0.492
myosin light chain kinase(MYLK)	-1.06	1.06	0.578
cyclin dependent kinase 16(CDK16)	-1.06	1.01	0.310
nuclear factor kappa B subunit 2(NFKB2)	-1.06	1.03	0.641
transmembrane protein 74(TMEM74)	-1.06	1.06	0.570
eukaryotic translation initiation factor 2 alpha kinase 3(EIF2AK3)	-1.06	1.15	0.687
kelch like family member 1(KLHL1)	-1.06	1.01	0.281
protein kinase C gamma(PRKCG)	-1.06	1.07	0.314
3-phosphoinositide dependent protein kinase 1(PDPK1)	-1.06	1.04	0.653
keratin 83(KRT83)	-1.06	1.24	0.687
ubiquitin conjugating enzyme E2 J2(UBE2J2)	-1.06	1.07	0.296
clock circadian regulator(CLOCK)	-1.06	1.06	0.399

clathrin light chain A(CLTA)	-1.06	1.08	0.329
mitogen-activated protein kinase 8(MAPK8)	-1.07	1.13	0.652
autophagy related 13(ATG13)	-1.07	1.05	0.432
autophagy related 13(ATG13)	-1.07	1.05	0.432
mitogen-activated protein kinase 10(MAPK10)	-1.07	1.03	0.073
solute carrier family 1 member 7(SLC1A7)	-1.07	1.12	0.455
VPS41, HOPS complex subunit(VPS41)	-1.07	1.04	0.093
lysosomal associated membrane protein 1(LAMP1)	-1.07	1.05	0.492
protein phosphatase 1 catalytic subunit alpha(PPP1CA)	-1.07	1.08	0.328
TNF receptor associated factor 4(TRAF4)	-1.07	1.07	0.229
cathepsin D(CTSD)	-1.07	1.03	0.235
transforming growth factor beta receptor associated protein 1(TGFBRAP1)	-1.08	1.04	0.164
pyrophosphatase (inorganic) 2(PPA2)	-1.08	1.09	0.526
ubiquitin conjugating enzyme E2 L3(UBE2L3)	-1.08	1.01	0.060
ubiquitin conjugating enzyme E2 A(UBE2A)	-1.08	1.05	0.514
calpain small subunit 1(CAPNS1)	-1.08	1.03	0.156
TNFRSF1A associated via death domain(TRADD)	-1.08	1.02	0.089
glial fibrillary acidic protein(GFAP)	-1.08	1.06	0.198
mannosidase alpha class 2B member 1(MAN2B1)	-1.08	1.10	0.474
TNF receptor associated factor 1(TRAF1)	-1.08	1.19	0.838
hypoxia inducible factor 1 alpha subunit(HIF1A)	-1.08	1.03	0.558
exocyst complex component 3 like 4(EXOC3L4)	-1.08	1.04	0.152
RAB1B, member RAS oncogene family(RAB1B)	-1.08	1.03	0.108
calpain 5(CAPN5)	-1.08	1.06	0.273
protein phosphatase 4 catalytic subunit(PPP4C)	-1.08	1.05	0.098
calpain 11(CAPN11)	-1.08	1.07	0.478
mitogen-activated protein kinase 15(MAPK15)	-1.08	1.03	0.218
golgi associated PDZ and coiled-coil motif containing(GOPC)	-1.08	1.07	0.332
adaptor related protein complex 2 sigma 1 subunit(AP2S1)	-1.08	1.02	0.009
pyrophosphatase (inorganic) 1(PPA1)	-1.08	1.02	0.162
solute carrier family 1 member 1(SLC1A1)	-1.08	1.11	0.436
erb-b2 receptor tyrosine kinase 4(ERBB4)	-1.09	1.07	0.477
RAB guanine nucleotide exchange factor 1(RABGEF1)	-1.09	1.11	0.369
protein kinase cAMP-activated catalytic subunit alpha(PRKACA)	-1.09	1.05	0.184

aryl hydrocarbon receptor nuclear translocator like(ARNTL)	-1.09	1.02	0.422
sorting nexin 4(SNX4)	-1.09	1.10	0.339
sorting nexin 4(SNX4)	-1.09	1.10	0.339
syntaxin 8(STX8)	-1.09	1.03	0.107
YME1 like 1 ATPase(YME1L1)	-1.09	1.09	0.428
CUGBP, Elav-like family member 1(CELF1)	-1.09	1.04	0.474
UDP glucuronosyltransferase family 2 member B7(UGT2B7)	-1.09	1.06	0.468
VPS18, CORVET/HOPS core subunit(VPS18)	-1.09	1.08	0.374
clathrin heavy chain(CLTC)	-1.09	1.04	0.073
TNF receptor associated factor 5(TRAF5)	-1.09	1.10	0.541
phosphatidylinositol-4,5-bisphosphate 3-kinase catalytic subunit delta(PIK3CD)	-1.09	1.09	0.289
peroxisomal biogenesis factor 5(PEX5)	-1.09	1.04	0.392
RAB10, member RAS oncogene family(RAB10)	-1.09	1.09	0.244
short coiled-coil protein(SCOC)	-1.10	1.05	0.167
vimentin(VIM)	-1.10	1.05	0.149
macrophage stimulating 1(MST1)	-1.10	1.07	0.584
RAB7A, member RAS oncogene family(RAB7A)	-1.10	1.03	0.030
trafficking protein particle complex 2(TRAPPC2)	-1.10	1.24	0.528
Rho GTPase activating protein 10(ARHGAP10)	-1.10	1.01	0.232
voltage dependent anion channel 2(VDAC2)	-1.10	1.05	0.291
adaptor related protein complex 3 mu 1 subunit(AP3M1)	-1.10	1.08	0.355
nuclear VCP-like(NVL)	-1.10	1.05	0.642
adaptor related protein complex 1 sigma 1 subunit(AP1S1)	-1.10	1.10	0.332
protein kinase C eta(PRKCH)	-1.10	1.05	0.418
SH3 domain containing GRB2 like endophilin B1(SH3GLB1)	-1.10	1.10	0.480
myosin light chain kinase 3(MYLK3)	-1.10	1.06	0.303
SHC adaptor protein 1(SHC1)	-1.10	1.03	0.376
voltage dependent anion channel 1(VDAC1)	-1.10	1.07	0.407
microtubule associated protein 1 light chain 3 beta(MAP1LC3B)	-1.10	1.06	0.308
solute carrier family 7 member 5(SLC7A5)	-1.10	1.29	0.931
Protein Phosphatase 2 Phosphatase Activator (PPP2R4)	-1.10	1.11	0.358
autophagy related 12(ATG12)	-1.10	1.03	0.026
autophagy related 12(ATG12)	-1.10	1.03	0.026
autophagy related 12(ATG12)	-1.10	1.03	0.026

ubiquitin conjugating enzyme E2 H(UBE2H)	-1.11	1.05	0.079
HRas proto-oncogene, GTPase(HRAS)	-1.11	1.06	0.195
tensin 3(TNS3)	-1.11	1.03	0.197
RAB5B, member RAS oncogene family(RAB5B)	-1.11	1.06	0.098
kelch like family member 17(KLHL17)	-1.11	1.04	0.052
WD repeat domain 3(WDR3)	-1.11	1.08	0.323
protein kinase, DNA-activated, catalytic polypeptide(PRKDC)	-1.11	1.00	0.251
spermatogenesis associated 5 like 1(SPATA5L1)	-1.11	1.09	0.228
secretion associated Ras related GTPase 1A(SAR1A)	-1.11	1.04	0.076
BCL2 associated athanogene 3(BAG3)	-1.11	1.12	0.491
dynamin 3(DNM3)	-1.11	1.07	0.764
active BCR-related(ABR)	-1.11	1.05	0.046
heat shock protein family A (Hsp70) member 2(HSPA2)	-1.12	1.06	0.728
vesicle associated membrane protein 7(VAMP7)	-1.12	1.13	0.305
ubiquitin B(UBB)	-1.12	1.12	0.291
exocyst complex component 5(EXOC5)	-1.12	1.09	0.263
Ras related GTP binding C(RRAGC)	-1.12	1.02	0.003
ret proto-oncogene(RET)	-1.12	1.10	0.325
adaptor related protein complex 2 mu 1 subunit(AP2M1)	-1.12	1.04	0.219
mitogen-activated protein kinase kinase kinase 6(MAP3K6)	-1.12	1.01	0.270
transcription factor EB(TFEB)	-1.12	1.05	0.070
RAS like proto-oncogene B(RALB)	-1.12	1.11	0.408
myeloid differentiation primary response 88(MYD88)	-1.12	1.04	0.142
eukaryotic translation initiation factor 2 alpha kinase 4(EIF2AK4)	-1.12	1.06	0.121
phosphatidylinositol-3,4,5-trisphosphate dependent Rac exchange factor 2(PREX2)	-1.12	1.03	0.149
aryl hydrocarbon receptor nuclear translocator 2(ARNT2)	-1.12	1.05	0.502
Ras related GTP binding D(RRAGD)	-1.12	1.06	0.174
trafficking protein particle complex 1(TRAPPC1)	-1.12	1.06	0.047
calcium/calmodulin dependent protein kinase kinase 2(CAMKK2)	-1.13	1.09	0.232
lipin 1(LPIN1)	-1.13	1.06	0.295
cyclin dependent kinase 5(CDK5)	-1.13	1.07	0.503
syntaxin 17(STX17)	-1.13	1.06	0.068
eukaryotic translation initiation factor 2 subunit alpha(EIF2S1)	-1.13	1.08	0.274
NUAK family kinase 1(NUAK1)	-1.13	1.07	0.133

UDP glucuronosyltransferase family 2 member A1 complex locus(UGT2A1)	-1.13	1.02	0.087
EPH receptor B1(EPHB1)	-1.13	1.11	0.371
phosphatidylinositol-4,5-bisphosphate 3-kinase catalytic subunit beta(PIK3CB)	-1.13	1.12	0.414
cyclin dependent kinase like 5(CDKL5)	-1.14	1.06	0.080
cyclin dependent kinase 2(CDK2)	-1.14	1.05	0.305
alkaline phosphatase, liver/bone/kidney(ALPL)	-1.14	1.12	0.266
inositol 1,4,5-trisphosphate receptor type 1(ITPR1)	-1.15	1.07	0.092
calpain 14(CAPN14)	-1.15	1.04	0.151
protein phosphatase 2 regulatory subunit B'delta(PPP2R5D)	-1.15	1.01	0.091
insulin receptor substrate 4(IRS4)	-1.15	1.04	0.013
apoptotic peptidase activating factor 1(APAF1)	-1.15	1.05	0.110
adaptor related protein complex 3 delta 1 subunit(AP3D1)	-1.15	1.06	0.244
ectopic P-granules autophagy protein 5 homolog(EPG5)	-1.15	1.06	0.081
phospholipase C gamma 1(PLCG1)	-1.15	1.01	0.196
ADP ribosylation factor 6(ARF6)	-1.15	1.04	0.016
SHC adaptor protein 2(SHC2)	-1.15	1.08	0.299
keratin 73(KRT73)	-1.15	1.13	0.203
actin beta(ACTB)	-1.15	1.04	0.108
spermatogenesis associated 5(SPATA5)	-1.16	1.12	0.382
sorting nexin 1(SNX1)	-1.16	1.07	0.160
SH3 domain containing GRB2 like 1, endophilin A2(SH3GL1)	-1.16	1.13	0.400
vesicle associated membrane protein 3(VAMP3)	-1.16	1.03	0.055
ABL proto-oncogene 2, non-receptor tyrosine kinase(ABL2)	-1.16	1.18	0.493
protein kinase C epsilon(PRKCE)	-1.16	1.09	0.237
RAB1A, member RAS oncogene family(RAB1A)	-1.16	1.05	0.028
keratin 74(KRT74)	-1.17	1.18	0.462
SID1 transmembrane family member 2(SIDT2)	-1.17	1.08	0.661
sestrin 1(SESN1)	-1.17	1.07	0.093
NLR family pyrin domain containing 5(NLRP5)	-1.17	1.08	0.190
REL proto-oncogene, NF-kB subunit(REL)	-1.17	1.04	0.087
alkaline phosphatase, placental like 2(ALPPL2)	-1.17	1.07	0.154
salt inducible kinase 2(SIK2)	-1.17	1.05	0.010
phosphatidylinositol-4,5-bisphosphate 3-kinase catalytic subunit gamma(PIK3CG)	-1.17	1.04	0.239
autophagy and beclin 1 regulator 1(AMBRA1)	-1.18	1.06	0.025

syntaxin 7(STX7)	-1.18	1.03	0.007
ankyrin repeat domain 28(ANKRD28)	-1.18	1.17	0.306
autophagy related 9B(ATG9B)	-1.18	1.11	0.101
phosphatidylinositol-4-phosphate 3-kinase catalytic subunit type 2 alpha(PIK3C2A)	-1.18	1.02	0.020
actin, alpha 1, skeletal muscle(ACTA1)	-1.18	1.04	0.023
lysosomal trafficking regulator(LYST)	-1.19	1.08	0.062
keratin 5(KRT5)	-1.19	1.12	0.122
mitogen-activated protein kinase associated protein 1(MAPKAP1)	-1.19	1.02	0.003
insulin like growth factor 1 receptor(IGF1R)	-1.19	1.07	0.071
tumor protein p53 inducible nuclear protein 2(TP53INP2)	-1.19	1.07	0.066
BCL2 like 1(BCL2L1)	-1.20	1.04	0.139
myotubularin related protein 2(MTMR2)	-1.20	1.12	0.194
casein kinase 1 alpha 1 like(CSNK1A1L)	-1.20	1.06	0.067
protein kinase AMP-activated non-catalytic subunit beta 2(PRKAB2)	-1.20	1.05	0.012
POC1 centriolar protein A(POC1A)	-1.20	1.01	0.006
ribosomal protein S6 kinase A3(RPS6KA3)	-1.20	1.11	0.100
protein arginine methyltransferase 3(PRMT3)	-1.20	1.07	0.384
exocyst complex component 6B(EXOC6B)	-1.20	1.06	0.148
adaptor related protein complex 1 sigma 3 subunit(AP1S3)	-1.21	1.08	0.171
exocyst complex component 6(EXOC6)	-1.21	1.09	0.089
protein kinase C delta(PRKCD)	-1.21	1.07	0.018
LPS responsive beige-like anchor protein(LRBA)	-1.21	1.02	0.069
autophagy related 10(ATG10)	-1.22	1.02	0.007
autophagy related 10(ATG10)	-1.22	1.02	0.007
WD repeat domain, phosphoinositide interacting 1(WIPI1)	-1.22	1.08	0.160
phospholipase D1(PLD1)	-1.22	1.05	0.072
phospholipase D1(PLD1)	-1.22	1.05	0.072
kelch like family member 20(KLHL20)	-1.22	1.02	0.014
neuroblastoma RAS viral oncogene homolog(NRAS)	-1.22	1.14	0.118
TRAF2 and NCK interacting kinase(TNIK)	-1.23	1.18	0.452
MX dynamin like GTPase 2(MX2)	-1.23	1.12	0.136
calcium/calmodulin dependent protein kinase II delta(CAMK2D)	-1.23	1.00	0.001
microtubule associated protein 1 light chain 3 gamma(MAP1LC3C)	-1.23	1.10	0.040
serine/threonine kinase 24(STK24)	-1.23	1.02	0.007

SHC adaptor protein 3(SHC3)	-1.23	1.09	0.254
transmembrane protein 173(TMEM173)	-1.24	1.04	0.505
tensin 1(TNS1)	-1.24	1.19	0.396
neurobeachin(NBEA)	-1.24	1.09	0.079
solute carrier family 7 member 6(SLC7A6)	-1.25	1.09	0.138
phospholipase C delta 1(PLCD1)	-1.25	1.08	0.172
ARP3 actin related protein 3 homolog(ACTR3)	-1.25	1.07	0.009
FGR proto-oncogene, Src family tyrosine kinase(FGR)	-1.25	1.04	0.247
Cdc42 guanine nucleotide exchange factor 9(ARHGEF9)	-1.25	1.06	0.015
mitogen-activated protein kinase kinase 1(MAP2K1)	-1.25	1.02	0.007
protein phosphatase 2 scaffold subunit Abeta(PPP2R1B)	-1.25	1.13	0.161
endothelial PAS domain protein 1(EPAS1)	-1.25	1.05	0.154
keratin 6B(KRT6B)	-1.25	1.20	0.198
mitogen-activated protein kinase kinase kinase kinase 3(MAP4K3)	-1.26	1.06	0.071
GLI pathogenesis related 2(GLIPR2)	-1.26	1.06	0.067
solute carrier family 7 member 8(SLC7A8)	-1.26	1.07	0.024
solute carrier family 1 member 4(SLC1A4)	-1.28	1.10	0.435
actin gamma 1(ACTG1)	-1.29	1.06	0.009
GABA type A receptor associated protein like 2(GABARAPL2)	-1.29	1.06	0.009
vacuolar protein sorting 13 homolog A(VPS13A)	-1.29	1.07	0.018
keratin 6C(KRT6C)	-1.29	1.04	0.166
parkin RBR E3 ubiquitin protein ligase(PARK2)	-1.29	1.03	0.043
isoleucyl-tRNA synthetase(IARS)	-1.30	1.27	0.338
Jun proto-oncogene, AP-1 transcription factor subunit(JUN)	-1.30	1.09	0.190
protein phosphatase 2 regulatory subunit B'alpha(PPP2R5A)	-1.31	1.10	0.076
TBC1 domain family member 4(TBC1D4)	-1.31	1.07	0.111
cyclin dependent kinase 18(CDK18)	-1.31	1.09	0.268
protein phosphatase 2 scaffold subunit Aalpha(PPP2R1A)	-1.33	1.04	0.036
protein kinase AMP-activated catalytic subunit alpha 1(PRKAA1)	-1.34	1.07	0.017
eukaryotic translation initiation factor 2 alpha kinase 2(EIF2AK2)	-1.34	1.07	0.020
calcium/calmodulin dependent protein kinase II beta(CAMK2B)	-1.34	1.13	0.058
solute carrier family 1 member 6(SLC1A6)	-1.34	1.09	0.119
autophagy related 4A cysteine peptidase(ATG4A)	-1.35	1.16	0.082
WDFY family member 4(WDFY4)	-1.35	1.04	0.036

mitofusin 1(MFN1)	-1.35	1.13	0.080
mitogen-activated protein kinase kinase kinase 14(MAP3K14)	-1.35	1.06	0.034
spermatogenesis associated 13(SPATA13)	-1.35	1.04	0.201
eukaryotic translation initiation factor 4E(EIF4E)	-1.35	1.05	0.125
phosphatidylinositol-4-phosphate 5-kinase type 1 beta(PIP5K1B)	-1.36	1.11	0.049
aurora kinase A(AURKA)	-1.36	1.04	0.030
myotubularin related protein 4(MTMR4)	-1.36	1.04	0.029
mitogen-activated protein kinase kinase kinase kinase 2(MAP4K2)	-1.37	1.04	0.010
mitogen-activated protein kinase kinase 3(MAP2K3)	-1.38	1.09	0.058
heat shock protein family A (Hsp70) member 5(HSPA5)	-1.40	1.10	0.036
protein kinase cAMP-activated catalytic subunit beta(PRKACB)	-1.41	1.05	0.012
sestrin 3(SESN3)	-1.47	1.34	0.414
BCL2, apoptosis regulator(BCL2)	-1.48	1.11	0.039
mitogen-activated protein kinase kinase kinase kinase 5(MAP4K5)	-1.48	1.12	0.045
pyruvate dehydrogenase kinase 1(PDK1)	-1.51	1.23	0.072
BCL2 interacting protein 3(BNIP3)	-1.53	1.26	0.077
sphingomyelin phosphodiesterase 1(SMPD1)	-1.54	1.09	0.011
phosphatidylinositol-4-phosphate 3-kinase catalytic subunit type 2 beta(PIK3C2B)	-1.55	1.11	0.269
fibroblast growth factor receptor 1(FGFR1)	-1.60	1.15	0.294
KIAA0226L	-1.70	1.18	0.085
protein kinase C alpha(PRKCA)	-1.71	1.09	0.012
E2F transcription factor 1(E2F1)	-1.75	1.19	0.029
ankyrin 2(ANK2)	-1.78	1.26	0.035
keratin 79(KRT79)	-2.02	1.67	0.147
epsin 2(EPN2)	-2.10	1.09	0.001
myotubularin related protein 1(MTMR1)	-2.16	1.04	0.010
protein phosphatase 2 regulatory subunit B''alpha(PPP2R3A)	-2.35	1.10	0.091
maternal embryonic leucine zipper kinase(MELK)	-2.92	1.36	0.009
cyclin dependent kinase 1(CDK1)	-3.03	1.46	0.027

* Fold change (linear) in the expression of each gene in ABI-Mac compared to that in AB-Mac
